# Utility of a Smartphone-Based Clinical Decision Support System for Pressure Ulcer Management by Physicians: Randomized Crossover Pilot Study

**DOI:** 10.2196/85452

**Published:** 2026-03-23

**Authors:** Takahiro Ito, Takanobu Hirosawa, Arisa Hayashi, Masashi Yokose, Taro Shimizu

**Affiliations:** 1 Department of Diagnostic and Generalist Medicine Dokkyo Medical University Tochigi Japan; 2 Satsuki Home Clinic Tochigi Japan

**Keywords:** clinical decision support system, pressure ulcer, wound healing, ointment, wound dressing, general internal medicine

## Abstract

**Background:**

Clinical decision support systems (CDSSs) are widely used in various health care settings. In Japan, pressure ulcers are becoming a major concern in an aging society due to their increasing prevalence. However, management is often handled by nonspecialists in wound care due to regional disparities in specialist availability.

**Objective:**

To provide support for nonspecialists in wound care, we developed a prototype smartphone-based CDSS for pressure ulcer management. The system prompts users to answer questions about the wound's condition and recommends appropriate ointments and wound dressings by using a safety-first approach. This study aims to evaluate the utility of this system.

**Methods:**

We conducted a randomized crossover pilot study involving 28 general internal medicine (GIM) physicians. Participants were randomly assigned to group A (intervention-control) or group B (control-intervention). Participants evaluated 10 standardized pressure ulcer photographs and selected the most appropriate ointment and wound dressing for each. The unit of analysis was the individual response to each question (N=280 total observations). We used generalized estimating equations with an exchangeable correlation structure to account for within-subject clustering and adjust for potential period and sequence effects.

**Results:**

The overall correct response rate during the intervention phase was significantly higher than that during the control phase (49.3% vs 4.3%, respectively). After adjusting for clustering and crossover biases, the use of CDSS was associated with a 29.1-fold increase in the odds of a correct response (95% CI 8.2-103; *P*<.001). Secondary analyses revealed significant improvements in ointment selection (adjusted odds ratio [aOR] 2.4, 95% CI 1.5-3.8; *P*<.001) and wound dressing selection (aOR 8.9, 95% CI 4.9-16.1; *P*<.001). However, no significant period (*P*=.11) or sequence (*P*=.25) effects were observed for the primary outcome.

**Conclusions:**

The prototype CDSS improved the accuracy of treatment decisions made by GIM physicians in a pilot study that used photographs and fixed options. Within the parameters of this investigation, CDSS effectively guided participants toward standardized, safety-oriented choices as defined by our scoring criteria.

**Trial Registration:**

UMIN Clinical Trials Registry UMIN000057294; https://tinyurl.com/36a6vvah

## Introduction

### Background

Clinical decision support systems (CDSSs) are software programs that integrate individual patient characteristics with a computerized knowledge base to generate patient-specific assessments or recommendations. These assessments and recommendations are designed to support physicians or patients in making clinical decisions [1]. Some CDSSs are primarily used at the point of care, enabling physicians to combine their expertise with the information or recommendations provided by CDSS [2]. CDSSs can optimize patient care by issuing warnings, reminders, and suggestions during diagnosis, examination, and treatment [3]. Research has also demonstrated that CDSSs can improve adherence to clinical guidelines [4]. This finding is especially relevant considering the low compliance rates with clinical guidelines worldwide. CDSS development is currently underway across various medical settings [5-8].

### Epidemiological Burden of Pressure Ulcers

Pressure ulcers are a health problem worldwide that are common among inpatients and older adults. According to a systematic review and meta-analysis, the incidence of pressure ulcers in inpatients is approximately 1 out of 10 [9]. Furthermore, population-based studies have highlighted that pressure ulcers occur outside health care settings. For example, a study conducted in a rural area of Japan reported a prevalence rate of 20.3 per 1000 individuals older than 65 years, rising to 44.6 per 1000 among those older than 80 years [10]. These data indicate a growing burden of pressure ulcers in aging populations.

### Barriers to Clinical Guideline Adherence

Despite the recognized importance of pressure ulcer management, the implementation of current clinical practice guidelines is insufficient. Notably, some physicians report a lack of confidence in diagnosing and treating pressure ulcers [11]. Moreover, of the 107 family physicians in Canada who participated in the survey, only approximately 16% expressed confidence in managing leg ulcers. Of these 107 physicians, 90 answered the attitude questions; the concerns reported included a lack of evidence-based clinical practice guidelines for leg ulcer treatment (74/90, 82.2%), which could lead to a decline in care quality such as delayed wound healing [12].

### Current State of CDSS for Pressure Ulcers

In Europe, a CDSS for wound care, treatment, and management has been developed [13]. CDSS is designed to facilitate wound assessment and guide the selection of appropriate wound products from the clinical product formulary. This CDSS is at the point of care and across various settings, encompassing acute and primary care. Further, in Japan, the following projects are progressing: preparations for the development of a CDSS for home-visiting nurses in pressure ulcer management [14] and the development of an AI-integrated DESIGN-R scale evaluation process to enhance diagnostic consistency and accuracy [15]. Therefore, CDSS for pressure ulcer management is an area with significant room for further development.

### Local Context and Study Objectives

In Japan, pressure ulcer management faces the issue that, due to regional disparities in the distribution of plastic surgeons, pressure ulcers are not necessarily treated by wound care specialists [16]. To address this issue, we developed a prototype CDSS developed with reference to Japanese clinical guidelines, specifically optimized to guide nonspecialists in wound care toward standardized, risk-averse treatment pathways. The primary objective of this study was to evaluate whether general internal medicine (GIM) physicians using this CDSS could accurately select appropriate and specifically safe management options for pressure ulcers.

## Methods

### Study Setting

This study was a randomized crossover study. As the utility of the system has not yet been established, the study was designed as a pilot trial. This study was conducted by the Department of Diagnostic and Generalist Medicine (the Department of GIM) at Dokkyo Medical University Hospital, Tochigi, Japan, between March 2025 and May 2025. This study consisted of two major components: (1) the development of a CDSS for pressure ulcers and (2) the evaluation of the utility of CDSS. This study adhered to the CONSORT-EHEALTH (Consolidated Standards of Reporting Trials of Electronic and Mobile Health Applications and Online Telehealth) guidelines (Multimedia Appendix 1).

### Ethical Considerations

The research adhered strictly to the Declaration of Helsinki guidelines to ensure ethical conduct in human participant research. Ethics approval was obtained from the institutional review board at Dokkyo Medical University Hospital (R-89-7J). After a detailed explanation of the study, written informed consent was obtained from all participants before participation. The data were processed anonymously so that individuals cannot be identified. Participation was entirely voluntary, and participants could withdraw from the study at any time without penalty. No compensation or incentives were provided to the participants.

### Participant Recruitment

This study included older residents and faculty members from the Department of GIM at Dokkyo Medical University Hospital in 2025. Older residents and faculty members with clinical practice of more than 3 and 6 years after graduation, respectively, were included. The exclusion criteria were refusal to participate in this study or the presence of severe vision loss.

### Development of CDSS for Pressure Ulcers

**Figure 1:**
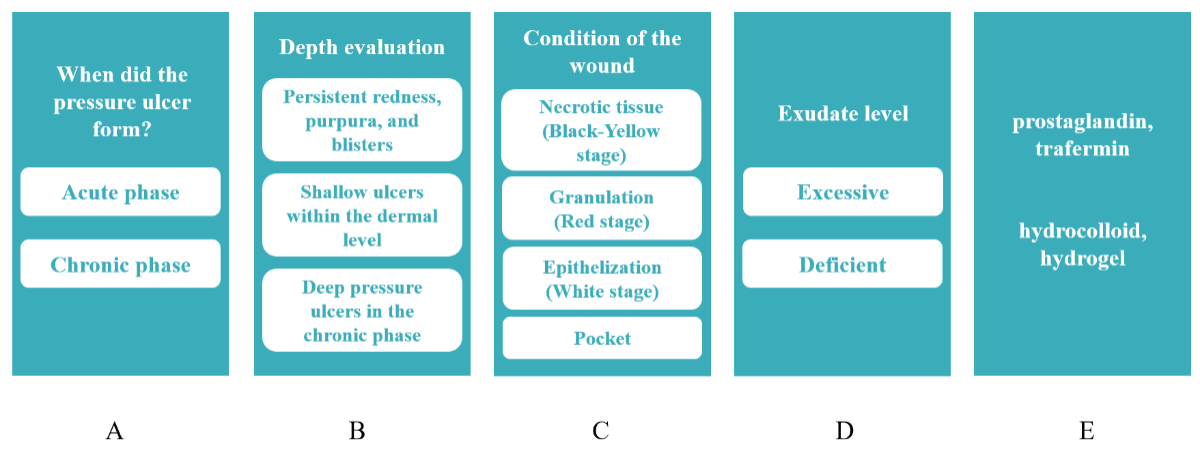
The clinical decision support system for pressure ulcers. (A) Selection screen for the phase when the pressure ulcer developed. (B) Selection screen for the depth evaluation in the chronic phase. (C) Selection screen for assessing the condition of deep pressure ulcers in the chronic phase. (D) Selection screen for the exudate level of deep pressure ulcers in the chronic phase. (E) Recommendation of ointments and wound dressings.

**Figure 2:**
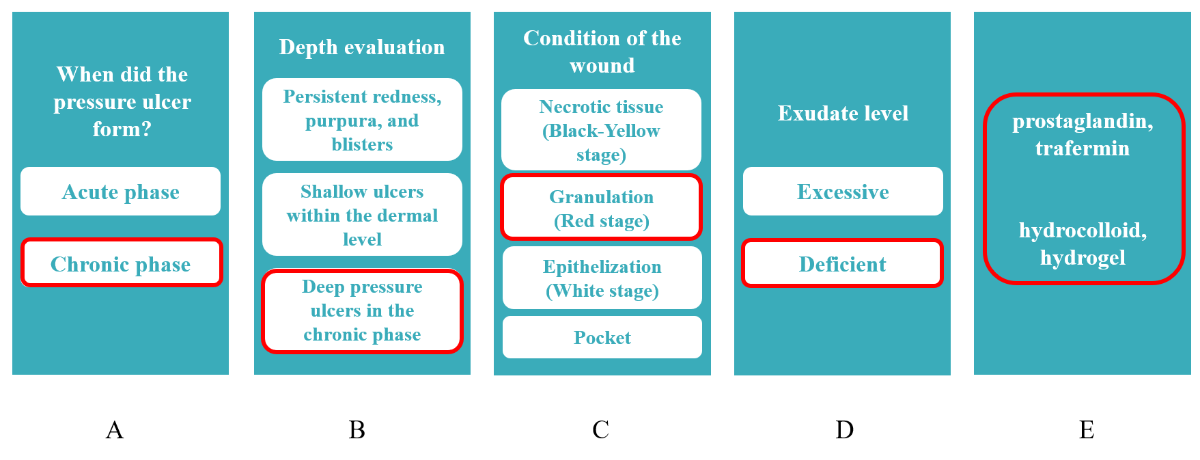
An example of how to use the clinical decision support system. Question 7: an ulcer with insufficient granulation tissue and minimal exudate. The selection buttons relevant to question 7 are highlighted in red. The screens transition sequentially from (A) to (E).

For this study, a prototype CDSS was created using the web-based no-code platform Canva [17]. CDSS is a prototype of a web application designed for use on mobile devices such as smartphones. The system has been developed to provide a framework for decision-making regarding the selection of ointments and wound dressings through a series of questions related to the patient’s pressure ulcers, as illustrated in Figure 1. Figure 2 presents an example of option selection in CDSS.

The decision logic of CDSS was constructed with a safety-first approach. Since the target users are nonspecialists in wound care who may not have access to surgical debridement, the algorithm was programmed to ensure safe management and strictly avoid high-risk options such as occluding infected sites, even when guidelines might offer conditional recommendations.

The system was developed based on two established clinical guidelines published by the *Japanese Dermatological Association* and the *Japanese Society of Pressure Ulcers* (JSPU) [18,19]. However, it was not designed to be an exact reproduction of these documents. Instead, we operationalized and simplified complex clinical algorithms, transforming them into structured decision trees tailored for GIM physicians who prioritize conservative, risk-averse decision-making.

According to the clinical algorithm in the Guidelines for the Diagnosis and Treatment of Pressure Ulcers, the first step is to diagnose the suspected lesion as a pressure ulcer [20]. The next step is to determine whether the pressure ulcer is in the acute or chronic phase. The subsequent management of pressure ulcers depends on their depth, and in particular, depth should be evaluated in chronic-phase ulcers. For severity classification and healing process quantification, the pressure ulcer staging scale DESIGN (depth, exudates, size, inflammation/infection, granulation, necrosis, and pocket) was developed by the JSPU Scientific Education Committee and published by the *Japanese Society for Pressure Ulcer Care* in 2002. The weighting of each of the 7 DESIGN criteria has been reviewed based on evidence, and the scale was published as DESIGN-R in 2008 [21].

According to the guidelines’ clinical algorithm for the treatment of chronic-phase deep pressure ulcers, the aim in the early treatment stages (black and yellow stages) is wound bed preparation based on the TIME concept. The TIME concept is based on the idea of evaluating factors that prevent wound healing from the viewpoint of tissue (T), infection or inflammation (I), moisture (M), and the wound edge (E) [22]. In the latter stages of treatment (red and white stages), the aim is to achieve moist wound healing [20]. In the black and yellow stages, the treatment of pressure ulcers involves removing the necrotic tissue, reducing the bacterial load, preventing wound drying, controlling excessive exudates, and treating pockets and wound edges. Subsequently, during both the red and white stages, maintaining the wound surface in a moist environment is crucial. CDSS suggests appropriate ointments and dressing materials based on users’ replies to prompts, which are generally structured per the process described above.

### Study Flow and Randomization

The randomization process was conducted electronically using Microsoft Excel and a computer-generated randomization table. By block randomization with the sealed envelope method, participants were randomly assigned to group A or B in a 1:1 ratio. This study used a crossover design, where all participants participated in both the intervention and control phases.

Participants were presented with pressure ulcer photographs and asked to select the most appropriate ointments and wound dressings from a set of options. Participants in group A initially participated in the intervention phase during period 1. In this phase, participants used CDSS to respond to the first set of questions (questions 1-5), followed by a washout period of at least 1 week. Subsequently, group A participants transitioned to the control phase during period 2, in which they answered the second set of questions (questions 6-10) without using CDSS. Participants in group B first completed the control phase during period 1, responding to questions 1-5 without using CDSS, followed by a washout period of at least 1 week. Group B participants then proceeded to the intervention phase during period 2, where CDSS was used to answer questions 6-10.

Responses were collected using an online form created with Microsoft Forms. The form contained the clinical photographs of pressure ulcers accompanied by multiple-choice response options. Participants could access the form by scanning a 2D barcode provided by the study coordinator or via a uniform resource locator shared through a group chat application.

The duration of the washout period was determined based on established theories of memory decay [23]. Additionally, in a previous study, a 1-week interval was implemented between the two phases to mitigate recall bias [24]. Consequently, a washout period of at least 1 week was implemented to mitigate carryover effects from the initial evaluation.

### Evaluation Materials

Participants evaluated the clinical photographs of pressure ulcers sourced from two publications by JSPU [25,26]. These publications are medically validated and authoritative sources that ensure the reliability and standardization of the referenced content. A total of 10 photographs were used, categorized into two sets (questions 1-5 and questions 6-10), with each set comprising 5 photographs. JSPU has formally provided permission for the use of these photographic materials in this investigation, contingent upon the requisite citation of the source. For all clinical scenarios, it was stipulated that surgical intervention was not available.

Regarding the selection of ointments, participants were instructed to select the most appropriate option from the following: (1) silver sulfadiazine; (2) povidone-iodine, cadexomer iodine, or iodoform; (3) povidone-iodine or bucladesine sodium; (4) povidone-iodine; (5) zinc oxide, dimethyl isopropyl azulene, or white petrolatum; and (6) prostaglandin or trafermin.

For the selection of wound dressings, participants were instructed to select the most appropriate option from the following: (1) silver-containing hydrofiber or silver-containing polyurethane foam, (2) silver-containing hydrofiber or silver-containing hydrocolloid, (3) silver-containing hydrocolloid or hydrogel, (4) polyurethane film or silver-containing hydrocolloid, (5) hydrogel, and (6) use of a dressing was considered inappropriate. Rather than standard non–silver-containing variants, silver-containing variants were used for several dressing categories to reflect contemporary clinical availability.

**Figure 3:**
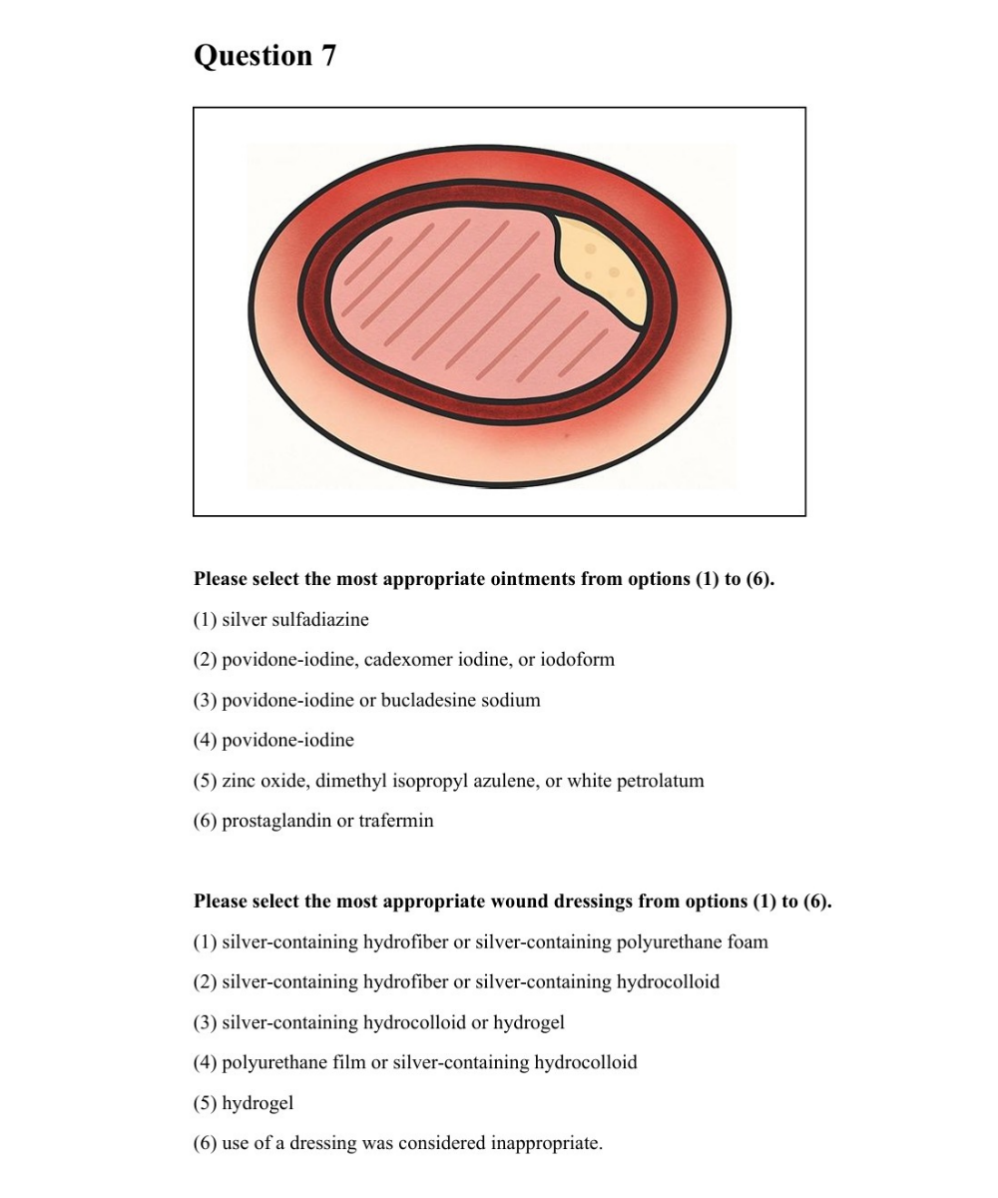
An illustrative image (used for question 7) of a sample version created to resemble the actual questionnaire used. This image was created with the assistance of ChatGPT (OpenAI), based on the authors’ instructions.

Question 7, for example, provides a second example of an ulcer with insufficient granulation tissue and minimal exudate (Figure 3). Topical treatments for such wounds include ointments such as prostaglandin or trafermin as well as wound dressings such as hydrocolloid or hydrogel. These treatments help to maintain adequate moisture on the wound surface. Therefore, the most appropriate choices are “prostaglandin or trafermin” ointment and “silver-containing hydrocolloid or hydrogel” wound dressing (Figure 2).

### Definition of Correct Answers and Scoring

The correct answers for ointments and wound dressings were defined by referencing the treatment algorithms and specific descriptions outlined in *Japanese Dermatological Association* and JSPU guidelines [18,19]. The clinical characteristics of each photograph and their corresponding correct answers are summarized in Table 1. For each question, one “most appropriate” option was selected for both the ointment and the dressing to ensure consistency in the statistical analysis. Regarding ointment selection, specifically for question 4, the source material [25] suggests that “silver sulfadiazine” and “povidone-iodine” are applicable; however, the guideline’s text explicitly recommends “povidone-iodine” [19]. To eliminate ambiguity and ensure statistical rigor, we established a strict scoring criterion that accepted only “povidone-iodine” as correct for this analysis (Figure 3).

**Table 1:** Details of the photographs used in each question and the correct answer.

	Details of the question	Ointments^a^	Wound dressings^b^
Question 1	Erosions or shallow ulcer involving damage limited to the dermis	(5) zinc oxide, dimethyl isopropyl azulene, or white petrolatum	(3) silver-containing hydrocolloid or hydrogel
Question 2	Ulcer with insufficient formation of healthy granulation tissue and a high amount of exudate	(3) povidone-iodine or bucladesine sodium	(1) silver-containing hydrofiber or silver-containing polyurethane foam
Question 3	Ulcer covered with dry black necrotic tissue, presenting minimal exudate and no signs of infection	(1) silver sulfadiazine	(5) hydrogel
Question 4	Ulcer with necrotic tissue in pockets but without signs of infection	(4) povidone-iodine	(6) use of a dressing was considered inappropriate
Question 5	Ulcer with well-developed healthy granulation tissue and abundant exudate	(3) povidone-iodine or bucladesine sodium	(1) silver-containing hydrofiber or silver-containing polyurethane foam
Question 6	Acute-stage pressure ulcer	(5) zinc oxide, dimethyl isopropyl azulene, or white petrolatum	(4) polyurethane film or silver-containing hydrocolloid
Question 7	Ulcer with insufficient granulation tissue and minimal exudate	(6) prostaglandin or trafermin	(3) silver-containing hydrocolloid or hydrogel
Question 8	Ulcer presenting with blisters	(5) zinc oxide, dimethyl isopropyl azulene, or white petrolatum	(4) polyurethane film or silver-containing hydrocolloid
Question 9	Ulcer covered with yellow necrotic tissue, abundant exudate, and clear signs of infection	(2) povidone-iodine, cadexomer iodine, or iodoform	(6) use of a dressing was considered inappropriate
Question 10	Ulcer with well-formed granulation tissue and minimal exudate	(6) prostaglandin or trafermin	(3) silver-containing hydrocolloid or hydrogel

^a^For ointment selection, participants were instructed to select the most appropriate option from the following: (1) silver sulfadiazine; (2) povidone-iodine, cadexomer iodine, or iodoform; (3) povidone-iodine or bucladesine sodium; (4) povidone-iodine; (5) zinc oxide, dimethyl isopropyl azulene, or white petrolatum; and (6) prostaglandin or trafermin.

^b^For the selection of wound dressings, participants were instructed to select the most appropriate option from the following: (1) silver-containing hydrofiber or silver-containing polyurethane foam, (2) silver-containing hydrofiber or silver-containing hydrocolloid, (3) silver-containing hydrocolloid or hydrogel, (4) polyurethane film or silver-containing hydrocolloid, (5) hydrogel, and (6) use of a dressing was considered inappropriate.

Furthermore, in clinical scenarios where the guidelines offered multiple options or weak recommendations, CDSS and the scoring criteria were designed to prioritize conservative, safety-oriented decision-making to minimize clinical risks for nonspecialists in wound care. Given the study's constraint that surgical intervention was unavailable, this safety-first approach distinguished between facilitating healing in stable wounds and avoiding high-risk occlusion in unstable wounds, especially in the dressing section.

Specifically, for an ulcer covered with dry, black, necrotic tissue, presenting minimal exudate and no signs of infection (question 3), “hydrogel” was the correct answer. Without surgical intervention, hydrogel is a conservative, low-risk method that facilitates autolytic debridement; the benefit of softening necrosis outweighs the minimal risk of complications.

Conversely, in scenarios where occlusion posed a significant clinical risk, such as an ulcer with necrotic tissue in pockets but without signs of infection (question 4) or an ulcer covered with yellow necrotic tissue, abundant exudate, and clear signs of infection (question 9), “use of a dressing was considered inappropriate” was the correct answer. Regarding question 4, sealing a cavity containing necrotic tissue carries a high risk of abscess formation or exacerbation of occult infection, especially given the constraints of this study, where surgical debridement was unavailable. Furthermore, regarding question 9, while the guideline offers “weak suggestion” (recommendation 2D) for silver-containing dressings in infected wounds, it also emphasizes that dressings are generally inappropriate in the presence of infection [19].

The “most appropriate” answers to the remaining questions were determined by ensuring they were supported by treatment algorithms or descriptive recommendations in at least one of the referenced guidelines [18,19].

### Data Collection and Outcome

Data regarding age, sex, and years since obtaining a medical degree were collected from all participants as baseline demographic data. This study used a questionnaire that required respondents to select the most appropriate ointments and wound dressings for treating pressure ulcers (Figure 3). The primary outcome was the proportion of participants who selected the correct answers for both ointment and wound dressing options. The secondary outcomes were comparisons of the average correct response rates for ointment selections and those for wound dressing selections. In order to evaluate the potential influence of learning or carryover effects despite the washout period, a sensitivity analysis was performed, restricted to period 1 data only. As an exploratory analysis, we investigated the utility of CDSS for identifying wound depth as deep or non-deep ulcers.

### Statistical Analysis

Statistical analyses were performed using R software (version 4.5.0; The R Foundation for Statistical Computing) for Windows. The unit of analysis was each individual question response (N=280, 28 physicians × 10 questions). To account for the crossover design and clustering of responses within physicians, generalized estimating equations (GEE) with a binomial distribution and logit link function were used. An exchangeable correlation structure was used to account for within-subject correlation. The model included the following fixed effects to assess intervention effects while adjusting for potential learning and carryover biases: treatment (intervention phase vs control phase), period (period 1 vs period 2), and sequence (group A vs group B). The intervention effect was reported as an adjusted odds ratio (aOR) with a 95% CI. Statistical significance was set at *P*<.05. The Mann-Whitney *U* test was used to compare continuous variables for baseline participant characteristics, which are presented as medians (IQRs). Fisher exact test was used to compare categorical and binary baseline participant characteristics, which are presented as counts (percentages).

## Results

### Baseline Characteristics

**Figure 4:**
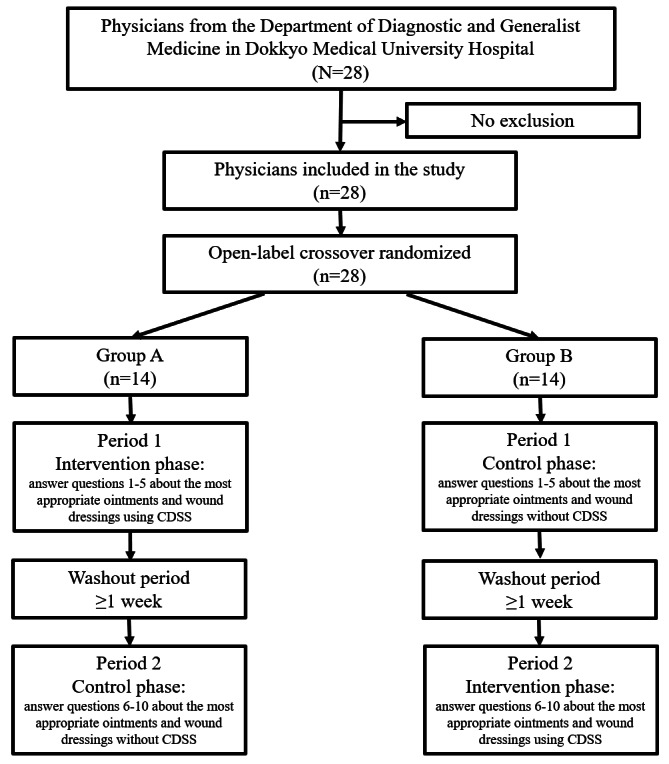
Flowchart of the study participants and allocation into groups. CDSS: clinical decision support system.

A total of 28 GIM physicians participated in this study (Figure 4). The median age of all participants was 32.2 (IQR 7.0) years; 20 participants (71.4%) were male, and the median number of years since graduation was 7.3 (IQR 6.0). Participant age (*P*=.39), sex (*P*=.21), and years since graduation (*P*=.25) did not differ significantly between group A and group B (Table 2).

**Table 2:** Baseline characteristics of the participants.

Variable	Group A (n=14)	Group B (n=14)	*P* value
Age (y), median (IQR)	32.8 (6.8)	31.6 (5.0)	.39^a^
Men, n (%)	12 (85.7)	8 (57.1)	.21^b^
Years after graduation (y), median (IQR)	8.1 (5.8)	6.5 (3.8)	.25^a^

^a^Mann-Whitney *U* test.

^b^Fisher exact test.

### Primary Outcomes

The primary outcome was the proportion of participants who correctly selected both the most appropriate ointment and the most appropriate wound dressing (Table 3). The overall correct response rate during the intervention phase was significantly higher than that during the control phase (49.3% vs 4.3%). After adjusting for clustering within physicians and potential crossover effects using GEE, CDSS use was associated with a 29.1-fold increase in the odds of a correct response (95% CI 8.2-103; *P*<.001). Regarding potential biases in the crossover design, there was no significant period effect (*P*=.11) or sequence effect (*P*=.25) for the primary outcome. This confirms the adequacy of the 1-week washout period.

### Secondary Outcomes

The secondary outcomes were comparisons of the average correct response rates for ointment and those for wound dressing (Table 3). Significant improvements were also observed in the secondary outcomes. The correct response rate for ointment selection increased from 42.9% in the control phase to 64.3% in the intervention phase (aOR 2.4, 95% CI 1.5-3.8; *P*<.001). Although a minor sequence effect was observed (*P*=.03), the intervention effect remained significant after adjustment. For wound dressing selection, the correct response rate increased from 15% in the control phase to 59.3% in the intervention phase (aOR 8.9, 95% CI 4.9-16.1; *P*<.001). A period effect was noted (*P*=.009), indicating a learning trend; however, the CDSS intervention remained the dominant predictor of success in the adjusted model.

**Table 3:** Effect of the clinical decision support system on pressure ulcer treatment selection (N=28).

	Intervention phase (%)^a^	Control phase (%)^a^	Adjusted odds ratio (95% CI)	*P* value
Total^b^	49.3	4.3	29.1 (8.2-103)	<.001
Ointments^c^	64.3	42.9	2.42 (1.5-3.8)	<.001
Wound dressings^c^	59.3	15	8.86 (4.9-16.1)	<.001

^a^Corresponding absolute values are not provided in the case of percentages that have been adjusted by the generalized estimating equations model.

^b^The primary outcome measure was the proportion of participants who correctly answered both the ointment and wound dressing selections. A total of 28 participants answered 5 questions during the intervention phase and another 5 during the control phase.

^c^The secondary outcome measures were comparisons of the average correct response rates for ointment selections and those for wound dressing selections. A total of 28 participants answered 5 questions during the intervention phase and another 5 during the control phase.

## Discussion

### Principal Results

This study yielded 3 main findings. First, the use of CDSS significantly increased the odds that GIM physicians would select the correct pressure ulcer treatment. The correct response rate increased from 4.3% to 49.3%, which is a 29.1-fold improvement (95% CI 8.2-103; *P*<.001). The extremely low correct response rate in the control phase underscores the inherent difficulty of appropriate treatment selection. This was likely influenced by our strict scoring criteria, which prioritized conservative, safety-oriented choices over multiple potential options. This result suggests that CDSS has the potential to assist physicians in cases of uncertainty about the appropriate treatment for pressure ulcers.

Second, CDSS demonstrated a particularly strong impact on wound dressing selection (aOR 8.9, 95% CI 4.9-16.1; *P*<.001) compared to ointment selection (aOR 2.4, 95% CI 1.5-3.8; *P*<.001). Notably, CDSS was particularly useful in identifying clinical scenarios in which dressings should be avoided. While some physicians lack knowledge about dressing properties [27], our system's design emphasizes the risks of occlusion in unstable wounds such as pockets or infection. This likely prevented hazardous treatment choices. Therefore, assistance in selecting wound dressings is likely to impact clinical practice in settings where consultation with specialists is not available immediately.

Third, we rigorously evaluated potential biases inherent in the crossover design by using GEE. For the primary outcome, neither the duration nor the sequence had a significant effect on the results, confirming the efficacy of the washout period. Although a significant period effect was observed in the dressing selection model (*P*=.009), indicating some spontaneous learning, the CDSS intervention effect remained significant after adjusting for this bias. This methodological approach ensures that the observed improvements are directly attributable to CDSS.

### Strengths

This study has 2 major strengths. First, the evaluation uses standardized clinical photographs from JSPU, which ensures reliability. Second, CDSS was designed to operationalize and optimize clinical algorithms. Unlike verbatim reproductions of guidelines, CDSS incorporates original, safety-first logic designed to minimize clinical risks such as abscess formation for nonspecialists in wound care working under the constraint of no surgical intervention. By converting complex and sometimes ambiguous guideline text into a structured, risk-averse decision tree, CDSS can standardize clinical decision-making and ensure patient safety in primary care settings.

### Limitations

This was a pilot study with 3 limitations. First, the study’s methodology was confined to the analysis of clinical photographs of pressure ulcers, excluding clinical courses, other physical examinations, and investigation results from real-world settings. Consequently, to substantiate the efficacy and legitimacy of CDSS for pressure ulcer management in clinical practice, subsequent studies involving actual patients, with careful consideration for patient safety, are required.

Second, the sample size was relatively small. This study was conducted by a department of GIM at a single institution. In the future, studies should include a more diverse sample of the target population. For example, home-visit physicians and physicians from other specialties working in inpatient wards should be included.

Third, the study used a forced-choice format based on a safety-first scoring system. This method may not fully capture the flexibility required in clinical practice. Although guidelines typically offer multiple options or weak recommendations, this study intentionally defined a single “most appropriate” answer to ensure statistical rigor and prioritize conservative management. In real-world settings, specialists might choose more varied treatments based on complex risk-benefit assessments that were not fully represented in the simplified scenarios of this pilot study.

### Comparison With Prior Work

There are some CDSSs for pressure ulcers that suggest treatment methods in addition to the one we have used that are currently available, for example, the WOUND COMPASS Clinical Support App [13]. However, to our knowledge, no study has compared the ability of physicians using CDSS to select appropriate treatments for pressure ulcers.

### Future Direction

The CDSS in this study was developed mainly by GIM physicians. In the future, collaboration with other specialists, including wound care experts, is required. To further improve the accuracy of CDSS, functions that support appropriate pressure ulcer assessment, as well as continuous skill training, should be incorporated. Furthermore, this CDSS is currently not approved by medical authorities. Achieving official approval through trials administered by medical authorities is crucial to establishing its credibility, safety, and adherence to recognized standards [28].

### Conclusions

This study was conducted as the first step in developing a CDSS for pressure ulcer management for GIM physicians who have limited opportunities to consult with wound care specialists. The prototype CDSS improved the accuracy of treatment decisions made by GIM physicians in a pilot study that used photographs and fixed options. Within the parameters of this investigation, CDSS effectively guided participants toward standardized, safety-oriented choices as defined by our scoring criteria.

## Appendix

Multimedia Appendix 1 CONSORT-EHEALTH checklist.

## Abbreviations

aOR: adjusted odds ratio
CDSS: clinical decision support system
CONSORT-EHEALTH: Consolidated Standards of Reporting Trials of Electronic and Mobile Health Applications and Online Telehealth
DESIGN: depth, exudates, size, inflammation/infection, granulation, necrosis, and pocket
GEE: generalized estimating equations
GIM: general internal medicine
JSPU: Japanese Society of Pressure Ulcers
TIME: tissue, infection/inflammation, moisture, edge
