# Supplementary material for: Utility of a Smartphone-Based Clinical Decision Support System for Pressure Ulcer Management by Physicians: Randomized Crossover Pilot Study
Source: JMIR Form Res. 2026 Mar 23;10:e85452. doi: 10.2196/85452 (PMC13054217; doi:10.2196/85452)
Supplement: Multimedia Appendix 1 [file formative_v10i1e85452_app1.pdf]

CONSORT-EHEALTH (V 1.6.1) - Submission/Publication Form

The CONSORT-EHEALTH checklist is intended for authors of randomized trials evaluating web-based and Internet-based applications/interventions, including mobile interventions, electronic games (incl multiplayer games), social media, certain telehealth applications, and other interactive and/or networked electronic applications. Some of the items (e.g. all subitems under item 5 - description of the intervention) may also be applicable for other study designs.

The goal of the CONSORT EHEALTH checklist and guideline is to be
a) a guide for reporting for authors of RCTs,
b) to form a basis for appraisal of an ehealth trial (in terms of validity)

CONSORT-EHEALTH items/subitems are MANDATORY reporting items for studies published in the Journal of Medical Internet Research and other journals / scientific societies endorsing the checklist.

Items numbered 1., 2., 3., 4a., 4b etc are original CONSORT or CONSORT-NPT (non-pharmacologic treatment) items.
Items with Roman numerals (i., ii, iii, iv etc.) are CONSORT-EHEALTH extensions/clarifications.

As the CONSORT-EHEALTH checklist is still considered in a formative stage, we would ask that you also RATE ON A SCALE OF 1-5 how important/useful you feel each item is FOR THE PURPOSE OF THE CHECKLIST and reporting guideline (optional).

Mandatory reporting items are marked with a red *.
In the textboxes, either copy & paste the relevant sections from your manuscript into this form - please include any quotes from your manuscript in QUOTATION MARKS,
or answer directly by providing additional information not in the manuscript, or elaborating on why the item was not relevant for this study.

YOUR ANSWERS WILL BE PUBLISHED AS A SUPPLEMENTARY FILE TO YOUR PUBLICATION IN JMIR AND ARE CONSIDERED PART OF YOUR PUBLICATION (IF ACCEPTED).
Please fill in these questions diligently. Information will not be copyedited, so please use proper spelling and grammar, use correct capitalization, and avoid abbreviations.

DO NOT FORGET TO SAVE AS PDF _AND_ CLICK THE SUBMIT BUTTON SO YOUR ANSWERS ARE IN OUR DATABASE !!!

Citation Suggestion (if you append the pdf as Appendix we suggest to cite this paper in the caption):
[illegible]

回答を編集しています。この URL を共有すると、他のユーザーもあなたの回答を編集できるようになります。

新しい回答を記入

[illegible]J Med Internet Res 2011;13(4):e126

URL: http://www.jmir.org/2011/4/e126/
doi: 10.2196/jmir.1923
PMID: 22209829

takahiro.ito10@gmail.com アカウントを切り替える

共有なし

再送信して保存

* 必須の質問です

Your name *

First Last

Takahiro Ito

Primary Affiliation (short), City, Country *

University of Toronto, Toronto, Canada

Department of Diagnostic and Generalist Medi

Your e-mail address *

abc@gmail.com

i-taka@dokkyomed.ac.jp

Title of your manuscript *

Provide the (draft) title of your manuscript.

Utility of A Smartphone-Based Clinical Decision Support System for Pressure Ulcer Management in Physicians: A Randomized Crossover Pilot Study

回答を編集しています。この URL を共有すると、他のユーザーもあなたの回答を編集できるようになります。

新しい回答を記入

Name of your App/Software/Intervention *

If there is a short and a long/alternate name, write the short name first and add the long name in brackets.

A smartphone-based clinical decision support

Evaluated Version (if any)

e.g. "V1", "Release 2017-03-01", "Version 2.0.27913"

回答を入力

Language(s) *

What language is the intervention/app in? If multiple languages are available, separate by comma (e.g. "English, French")

Japanese

URL of your Intervention Website or App

e.g. a direct link to the mobile app on app in appstore (itunes, Google Play), or URL of the website. If the intervention is a DVD or hardware, you can also link to an Amazon page.

回答を入力

URL of an image/screenshot (optional)

回答を入力

回答を編集しています。この URL を共有すると、他のユーザーもあなたの回答を編集できるようになります。 新しい回答を記入

Accessibility *

Can an enduser access the intervention presently?

- ◯ access is free and open
- ◯ access only for special usergroups, not open
- ◯ access is open to everyone, but requires payment/subscription/in-app purchases
- ◯ app/intervention no longer accessible
- ◉ その他: This information is not publicly available, but it will be disclosed upoı

Primary Medical Indication/Disease/Condition *

e.g. "Stress", "Diabetes", or define the target group in brackets after the condition, e.g. "Autism (Parents of children with)", "Alzheimers (Informal Caregivers of)"

Pressure ulcer

Primary Outcomes measured in trial *

comma-separated list of primary outcomes reported in the trial

The primary outcome was the proportion of pa

Secondary/other outcomes

Are there any other outcomes the intervention is expected to affect?

The secondary outcomes were comparisons of the correct response rates for ointment and for wound dressing options.

回答を編集しています。この URL を共有すると、他のユーザーもあなたの回答を編集できるようになります。 新しい回答を記入

Recommended "Dose" *

What do the instructions for users say on how often the app should be used?

- ( ) Approximately Daily
- ( ) Approximately Weekly
- ( ) Approximately Monthly
- ( ) Approximately Yearly
- (x) "as needed"
- ( ) その他:

Approx. Percentage of Users (starters) still using the app as recommended after 3 months *

- (x) unknown / not evaluated
- ( ) 0-10%
- ( ) 11-20%
- ( ) 21-30%
- ( ) 31-40%
- ( ) 41-50%
- ( ) 51-60%
- ( ) 61-70%
- ( ) 71%-80%
- ( ) 81-90%
- ( ) 91-100%
- ( ) その他:

回答を編集しています。この URL を共有すると、他のユーザーもあなたの回答を編集できるようになります。 新しい回答を記入

Overall, was the app/intervention effective? *

- (x) yes: all primary outcomes were significantly better in intervention group vs control
- ( ) partly: SOME primary outcomes were significantly better in intervention group vs control
- ( ) no statistically significant difference between control and intervention
- ( ) potentially harmful: control was significantly better than intervention in one or more outcomes
- ( ) inconclusive: more research is needed
- ( ) その他:

Article Preparation Status/Stage *

At which stage in your article preparation are you currently (at the time you fill in this form)

- ( ) not submitted yet - in early draft status
- (x) not submitted yet - in late draft status, just before submission
- ( ) submitted to a journal but not reviewed yet
- ( ) submitted to a journal and after receiving initial reviewer comments
- ( ) submitted to a journal and accepted, but not published yet
- ( ) published
- ( ) その他:

回答を編集しています。この URL を共有すると、他のユーザーもあなたの回答を編集できるようになります。 新しい回答を記入

Journal *

If you already know where you will submit this paper (or if it is already submitted), please provide the journal name (if it is not JMIR, provide the journal name under "other")

- ○ not submitted yet / unclear where I will submit this
- ○ Journal of Medical Internet Research (JMIR)
- ○ JMIR mHealth and UHealth
- ○ JMIR Serious Games
- ○ JMIR Mental Health
- ○ JMIR Public Health
- ◉ JMIR Formative Research
- ○ Other JMIR sister journal
- ○ その他:

Is this a full powered effectiveness trial or a pilot/feasibility trial? *

- ◉ Pilot/feasibility
- ○ Fully powered

Manuscript tracking number *

If this is a JMIR submission, please provide the manuscript tracking number under "other" (The ms tracking number can be found in the submission acknowledgement email, or when you login as author in JMIR. If the paper is already published in JMIR, then the ms tracking number is the four-digit number at the end of the DOI, to be found at the bottom of each published article in JMIR)

- ◉ no ms number (yet) / not (yet) submitted to / published in JMIR
- ○ その他:

回答を編集しています。この URL を共有すると、他のユーザーもあなたの回答を編集できるようになります。 新しい回答を記入

TITLE AND ABSTRACT

1a) TITLE: Identification as a randomized trial in the title

1a) Does your paper address CONSORT item 1a? *

I.e does the title contain the phrase "Randomized Controlled Trial"? (if not, explain the reason under "other")

- (x) yes
- ( ) その他:

1a-i) Identify the mode of delivery in the title

Identify the mode of delivery. Preferably use “web-based” and/or “mobile” and/or “electronic game” in the title. Avoid ambiguous terms like “online”, “virtual”, “interactive”. Use “Internet-based” only if Intervention includes non-web-based Internet components (e.g. email), use “computer-based” or “electronic” only if offline products are used. Use “virtual” only in the context of “virtual reality” (3-D worlds). Use “online” only in the context of “online support groups”. Complement or substitute product names with broader terms for the class of products (such as “mobile” or “smart phone” instead of “iphone”), especially if the application runs on different platforms.

	1	2	3	4	5	
subitem not at all important	( )	( )	( )	( )	( )	essential

Does your paper address subitem 1a-i? *

Copy and paste relevant sections from manuscript title (include quotes in quotation marks "like this" to indicate direct quotes from your manuscript), or elaborate on this item by providing additional information not in the ms, or briefly explain why the item is not applicable/relevant for your study

A Randomized Crossover Pilot Study

回答を編集しています。この URL を共有すると、他のユーザーもあなたの回答を編集できるようになります。 新しい回答を記入

1a-ii) Non-web-based components or important co-interventions in title

Mention non-web-based components or important co-interventions in title, if any (e.g., “with telephone support”).

	1	2	3	4	5	
subitem not at all important	○	○	○	○	○	essential

Does your paper address subitem 1a-ii?

Copy and paste relevant sections from manuscript title (include quotes in quotation marks "like this" to indicate direct quotes from your manuscript), or elaborate on this item by providing additional information not in the ms, or briefly explain why the item is not applicable/relevant for your study

回答を入力

1a-iii) Primary condition or target group in the title

Mention primary condition or target group in the title, if any (e.g., “for children with Type I Diabetes”) Example: A Web-based and Mobile Intervention with Telephone Support for Children with Type I Diabetes: Randomized Controlled Trial

	1	2	3	4	5	
subitem not at all important	○	○	○	○	○	essential

Does your paper address subitem 1a-iii? *

Copy and paste relevant sections from manuscript title (include quotes in quotation marks "like this" to indicate direct quotes from your manuscript), or elaborate on this item by providing additional information not in the ms, or briefly explain why the item is not applicable/relevant for your study

Utility of A Smartphone-Based Clinical Decision Support System for Pressure Ulcer Management in Physicians: A Randomized Crossover Pilot Study

回答を編集しています。この URL を共有すると、他のユーザーもあなたの回答を編集できるようになります。 新しい回答を記入

1b) ABSTRACT: Structured summary of trial design, methods, results, and conclusions

NPT extension: Description of experimental treatment, comparator, care providers, centers, and blinding status.

1b-i) Key features/functionalities/components of the intervention and comparator in the METHODS section of the ABSTRACT

Mention key features/functionalities/components of the intervention and comparator in the abstract. If possible, also mention theories and principles used for designing the site. Keep in mind the needs of systematic reviewers and indexers by including important synonyms. (Note: Only report in the abstract what the main paper is reporting. If this information is missing from the main body of text, consider adding it)

	1	2	3	4	5	
subitem not at all important	○	○	○	○	○	essential

Does your paper address subitem 1b-i? *

Copy and paste relevant sections from the manuscript abstract (include quotes in quotation marks "like this" to indicate direct quotes from your manuscript), or elaborate on this item by providing additional information not in the ms, or briefly explain why the item is not applicable/relevant for your study

Methods: In a randomized crossover pilot study, 28 General Internal Medicine (GIM) physicians were randomly assigned to Group A and Group B in a 1:1 ratio using block randomization with the sealed envelope method. Participants evaluated pressure ulcer photographs and answered questions requiring them to select both the most appropriate ointment and wound dressings. Participants in Group A first answered five multiple-choice questions as part of an intervention phase using the CDSS. After a washout period, the participants answered another five questions as part of a control phase without using the CDSS. Participants in Group B first participated in the control phase and then in the intervention phase. The primary outcome was the proportion of participants who selected both the correct ointment and wound dressing options. The secondary outcomes were comparisons of the correct response rates for ointment and for wound dressing options.

回答を編集しています。この URL を共有すると、他のユーザーもあなたの回答を編集できるようになります。 新しい回答を記入

1b-ii) Level of human involvement in the METHODS section of the ABSTRACT

Clarify the level of human involvement in the abstract, e.g., use phrases like “fully automated” vs. “therapist/nurse/care provider/physician-assisted” (mention number and expertise of providers involved, if any). (Note: Only report in the abstract what the main paper is reporting. If this information is missing from the main body of text, consider adding it)

	1	2	3	4	5	
subitem not at all important	○	○	○	○	○	essential

Does your paper address subitem 1b-ii?

Copy and paste relevant sections from the manuscript abstract (include quotes in quotation marks "like this" to indicate direct quotes from your manuscript), or elaborate on this item by providing additional information not in the ms, or briefly explain why the item is not applicable/relevant for your study

回答を入力

1b-iii) Open vs. closed, web-based (self-assessment) vs. face-to-face assessments in the METHODS section of the ABSTRACT

Mention how participants were recruited (online vs. offline), e.g., from an open access website or from a clinic or a closed online user group (closed usergroup trial), and clarify if this was a purely web-based trial, or there were face-to-face components (as part of the intervention or for assessment). Clearly say if outcomes were self-assessed through questionnaires (as common in web-based trials). Note: In traditional offline trials, an open trial (open-label trial) is a type of clinical trial in which both the researchers and participants know which treatment is being administered. To avoid confusion, use “blinded” or “unblinded” to indicated the level of blinding instead of “open”, as “open” in web-based trials usually refers to “open access” (i.e. participants can self-enrol). (Note: Only report in the abstract what the main paper is reporting. If this information is missing from the main body of text, consider adding it)

	1	2	3	4	5	
subitem not at all important	○	○	○	○	○	essential

回答を編集しています。この URL を共有すると、他のユーザーもあなたの回答を編集できるようになります。 新しい回答を記入

Does your paper address subitem 1b-iii?

Copy and paste relevant sections from the manuscript abstract (include quotes in quotation marks "like this" to indicate direct quotes from your manuscript), or elaborate on this item by providing additional information not in the ms, or briefly explain why the item is not applicable/relevant for your study

回答を入力

1b-iv) RESULTS section in abstract must contain use data

Report number of participants enrolled/assessed in each group, the use/uptake of the intervention (e.g., attrition/adherence metrics, use over time, number of logins etc.), in addition to primary/secondary outcomes. (Note: Only report in the abstract what the main paper is reporting. If this information is missing from the main body of text, consider adding it)

	1	2	3	4	5	
subitem not at all important	○	○	○	○	○	essential

Does your paper address subitem 1b-iv?

Copy and paste relevant sections from the manuscript abstract (include quotes in quotation marks "like this" to indicate direct quotes from your manuscript), or elaborate on this item by providing additional information not in the ms, or briefly explain why the item is not applicable/relevant for your study

回答を入力

回答を編集しています。この URL を共有すると、他のユーザーもあなたの回答を編集できるようになります。 新しい回答を記入

1b-v) CONCLUSIONS/DISCUSSION in abstract for negative trials

Conclusions/Discussions in abstract for negative trials: Discuss the primary outcome - if the trial is negative (primary outcome not changed), and the intervention was not used, discuss whether negative results are attributable to lack of uptake and discuss reasons. (Note: Only report in the abstract what the main paper is reporting. If this information is missing from the main body of text, consider adding it)

	1	2	3	4	5	
subitem not at all important	○	○	○	○	○	essential

Does your paper address subitem 1b-v?

Copy and paste relevant sections from the manuscript abstract (include quotes in quotation marks "like this" to indicate direct quotes from your manuscript), or elaborate on this item by providing additional information not in the ms, or briefly explain why the item is not applicable/relevant for your study

回答を入力

INTRODUCTION

2a) In INTRODUCTION: Scientific background and explanation of rationale

2a-i) Problem and the type of system/solution

Describe the problem and the type of system/solution that is object of the study: intended as stand-alone intervention vs. incorporated in broader health care program? Intended for a particular patient population? Goals of the intervention, e.g., being more cost-effective to other interventions, replace or complement other solutions? (Note: Details about the intervention are provided in “Methods” under 5)

	1	2	3	4	5	
subitem not at all important	○	○	○	○	○	essential

回答を編集しています。この URL を共有すると、他のユーザーもあなたの回答を編集できるようになります。 新しい回答を記入

Does your paper address subitem 2a-i? *

Copy and paste relevant sections from the manuscript (include quotes in quotation marks "like this" to indicate direct quotes from your manuscript), or elaborate on this item by providing additional information not in the ms, or briefly explain why the item is not applicable/relevant for your study

Therefore, we have developed a CDSS prototype to assist physicians in Japan who do not specialize in wound care, including General Internal Medicine (GIM) physicians. However, the utility of this CDSS has yet to be determined.

2a-ii) Scientific background, rationale: What is known about the (type of) system

Scientific background, rationale: What is known about the (type of) system that is the object of the study (be sure to discuss the use of similar systems for other conditions/diagnoses, if appropiate), motivation for the study, i.e. what are the reasons for and what is the context for this specific study, from which stakeholder viewpoint is the study performed, potential impact of findings [2]. Briefly justify the choice of the comparator.

	1	2	3	4	5	
subitem not at all important	○	○	○	○	○	essential

回答を編集しています。この URL を共有すると、他のユーザーもあなたの回答を編集できるようになります。 新しい回答を記入

Does your paper address subitem 2a-ii? *

Copy and paste relevant sections from the manuscript (include quotes in quotation marks "like this" to indicate direct quotes from your manuscript), or elaborate on this item by providing additional information not in the ms, or briefly explain why the item is not applicable/relevant for your study

For this study, a prototype CDSS was created using the web-based no-code platform Canva [15]. The CDSS is a prototype of a web application designed for use on mobile devices, such as smartphones. The system has been developed to provide a framework for decision-making regarding the selection of ointments and wound dressings, through a series of questions related to the patient's pressure ulcers, as illustrated in Figure 1. Figure 2 presents an example of option selection in the CDSS. The system's purpose is to supplement physicians' decision-making in various clinical settings, including the GIM setting. Two existing clinical guidelines were utilized to support Japanese physicians: the Guidelines for the Diagnosis and Treatment of Pressure Ulcers published by the Japanese Dermatological Association and the Guidelines for the Prevention and Management of Pressure Ulcers published by the Japanese Society of Pressure Ulcers (JSPU) [16,17].

According to the clinical algorithm in the Guidelines for the Diagnosis and Treatment of Pressure Ulcers, the first step is to diagnose the suspected lesion as a pressure ulcer [18]. The next step is to determine whether the pressure ulcer is in the acute or chronic phase. The subsequent management of pressure ulcers depends on their depth, and in particular, depth should be evaluated in chronic-phase ulcers. For severity classification and healing process quantification, the pressure ulcer staging scale DESIGN (depth, exudates, size, inflammation/infection, granulation, necrosis, and pocket) was developed by the JSPU Scientific Education Committee and published by the Japanese Society for Pressure Ulcer Care in 2002. The weighting of each of the seven DESIGN criteria has been reviewed based on evidence, and the scale was published as DESIGN-R in 2008 [19].

According to the Guidelines' clinical algorithm for the treatment of chronic-phase deep pressure ulcers, the aim in the early treatment stages (black and yellow stages) is wound bed preparation based on the TIME concept. The TIME concept is based on the idea of evaluating factors that prevent wound healing from the viewpoint of tissue (T), infection or inflammation (I), moisture (M), and the wound edge (E) [20]. In the latter stages of treatment (red and white stages), the aim is to achieve moist wound healing [18]. In the black and yellow stages, the treatment of pressure ulcers involves removing necrotic tissue, reducing the bacterial load, preventing wound drying, controlling excessive exudates, and treating pockets and wound edges. Subsequently, during both the red and white stages, maintaining the wound surface in a moist environment is crucial. The CDSS suggests appropriate ointments and dressing materials based on users' replies to prompts, which are generally structured per the process described above.

回答を編集しています。この URL を共有すると、他のユーザーもあなたの回答を編集できるようになります。 新しい回答を記入

Does your paper address CONSORT subitem 2b? *

Copy and paste relevant sections from the manuscript (include quotes in quotation marks "like this" to indicate direct quotes from your manuscript), or elaborate on this item by providing additional information not in the ms, or briefly explain why the item is not applicable/relevant for your study

The objective of the present study was to develop a CDSS and determine whether GIM physicians using this system can accurately select appropriate management for pressure ulcers.

METHODS

3a) Description of trial design (such as parallel, factorial) including allocation ratio

Does your paper address CONSORT subitem 3a? *

Copy and paste relevant sections from the manuscript (include quotes in quotation marks "like this" to indicate direct quotes from your manuscript), or elaborate on this item by providing additional information not in the ms, or briefly explain why the item is not applicable/relevant for your study

This study consisted of two major components: the development of a CDSS for pressure ulcers and the evaluation of the utility of the CDSS. This study was a randomized crossover study. As the utility of the system has not yet been established, the study was designed as a pilot trial.

3b) Important changes to methods after trial commencement (such as eligibility criteria), with reasons

回答を編集しています。この URL を共有すると、他のユーザーもあなたの回答を編集できるようになります。 新しい回答を記入

Does your paper address CONSORT subitem 3b? *

Copy and paste relevant sections from the manuscript (include quotes in quotation marks "like this" to indicate direct quotes from your manuscript), or elaborate on this item by providing additional information not in the ms, or briefly explain why the item is not applicable/relevant for your study

This study was not changed to methods after trial commencement (such as eligibility criteria), with reasons.

3b-i) Bug fixes, Downtimes, Content Changes

Bug fixes, Downtimes, Content Changes: ehealth systems are often dynamic systems. A description of changes to methods therefore also includes important changes made on the intervention or comparator during the trial (e.g., major bug fixes or changes in the functionality or content) (5-iii) and other “unexpected events” that may have influenced study design such as staff changes, system failures/downtimes, etc. [2].

	1	2	3	4	5	
subitem not at all important	○	○	○	○	○	essential

Does your paper address subitem 3b-i?

Copy and paste relevant sections from the manuscript (include quotes in quotation marks "like this" to indicate direct quotes from your manuscript), or elaborate on this item by providing additional information not in the ms, or briefly explain why the item is not applicable/relevant for your study

回答を入力

4a) Eligibility criteria for participants

回答を編集しています。この URL を共有すると、他のユーザーもあなたの回答を編集できるようになります。 新しい回答を記入

Does your paper address CONSORT subitem 4a? *

Copy and paste relevant sections from the manuscript (include quotes in quotation marks "like this" to indicate direct quotes from your manuscript), or elaborate on this item by providing additional information not in the ms, or briefly explain why the item is not applicable/relevant for your study

This study included senior residents and faculty members from the Department of GIM at Dokkyo Medical University Hospital in 2025. Senior residents and faculty members with clinical practice of more than 3 and 6 years after graduation, respectively, were included. The exclusion criteria were refusal to participate in this study or the presence of severe vision loss. After a detailed explanation of the study, written informed consent was obtained from all participants before participation.

4a-i) Computer / Internet literacy

Computer / Internet literacy is often an implicit “de facto” eligibility criterion - this should be explicitly clarified.

	1	2	3	4	5	
subitem not at all important	○	○	○	○	○	essential

Does your paper address subitem 4a-i?

Copy and paste relevant sections from the manuscript (include quotes in quotation marks "like this" to indicate direct quotes from your manuscript), or elaborate on this item by providing additional information not in the ms, or briefly explain why the item is not applicable/relevant for your study

回答を入力

回答を編集しています。この URL を共有すると、他のユーザーもあなたの回答を編集できるようになります。 新しい回答を記入

4a-ii) Open vs. closed, web-based vs. face-to-face assessments:

Open vs. closed, web-based vs. face-to-face assessments: Mention how participants were recruited (online vs. offline), e.g., from an open access website or from a clinic, and clarify if this was a purely web-based trial, or there were face-to-face components (as part of the intervention or for assessment), i.e., to what degree got the study team to know the participant. In online-only trials, clarify if participants were quasi-anonymous and whether having multiple identities was possible or whether technical or logistical measures (e.g., cookies, email confirmation, phone calls) were used to detect/prevent these.

	1	2	3	4	5	
subitem not at all important	○	○	○	○	○	essential

Does your paper address subitem 4a-ii? *

Copy and paste relevant sections from the manuscript (include quotes in quotation marks "like this" to indicate direct quotes from your manuscript), or elaborate on this item by providing additional information not in the ms, or briefly explain why the item is not applicable/relevant for your study

Participants were presented with pressure ulcer photographs and asked to select the most appropriate ointments and wound dressings from a set of options. Participants in Group A initially participated in the intervention phase during Period 1. In this phase, participants utilized the CDSS to respond to the first set of questions (Questions 1–5), followed by a washout period of at least 1 week. Subsequently, Group A participants transitioned to the control phase during Period 2, in which they answered the second set of questions (Questions 6–10) without using the CDSS. Participants in Group B first completed the control phase during Period 1, responding to Questions 1–5 without using the CDSS, followed by a washout period of at least 1 week. Group B participants then proceeded to the intervention phase during Period 2, where the CDSS was used to answer Questions 6–10. The study flow is shown in Figure 3.

4a-iii) Information giving during recruitment

Information given during recruitment. Specify how participants were briefed for recruitment and in the informed consent procedures (e.g., publish the informed consent documentation as appendix, see also item X26), as this information may have an effect on user self-selection, user expectation and may also bias results.

1	2	3	4	5

回答を編集しています。この URL を共有すると、他のユーザーもあなたの回答を編集できるようになります。 新しい回答を記入

Does your paper address subitem 4a-iii?

Copy and paste relevant sections from the manuscript (include quotes in quotation marks "like this" to indicate direct quotes from your manuscript), or elaborate on this item by providing additional information not in the ms, or briefly explain why the item is not applicable/relevant for your study

回答を入力

4b) Settings and locations where the data were collected

Does your paper address CONSORT subitem 4b? *

Copy and paste relevant sections from the manuscript (include quotes in quotation marks "like this" to indicate direct quotes from your manuscript), or elaborate on this item by providing additional information not in the ms, or briefly explain why the item is not applicable/relevant for your study

Responses were collected using an online form created with Microsoft Forms. The form contained clinical photographs of pressure ulcers accompanied by multiple-choice response options. Participants could access the form by scanning a 2-dimensional (2D) barcode provided by the study coordinator or via a Uniform Resource Locator shared through a group chat application.

4b-i) Report if outcomes were (self-)assessed through online questionnaires

Clearly report if outcomes were (self-)assessed through online questionnaires (as common in web-based trials) or otherwise.

	1	2	3	4	5	
subitem not at all important	○	○	○	○	○	essential

回答を編集しています。この URL を共有すると、他のユーザーもあなたの回答を編集できるようになります。 新しい回答を記入

Does your paper address subitem 4b-i? *

Copy and paste relevant sections from the manuscript (include quotes in quotation marks "like this" to indicate direct quotes from your manuscript), or elaborate on this item by providing additional information not in the ms, or briefly explain why the item is not applicable/relevant for your study

This study did not include report if outcomes were (self-)assessed through online questionnaires.

4b-ii) Report how institutional affiliations are displayed

Report how institutional affiliations are displayed to potential participants [on ehealth media], as affiliations with prestigious hospitals or universities may affect volunteer rates, use, and reactions with regards to an intervention.(Not a required item – describe only if this may bias results)

	1	2	3	4	5	
subitem not at all important	○	○	○	○	○	essential

Does your paper address subitem 4b-ii?

Copy and paste relevant sections from the manuscript (include quotes in quotation marks "like this" to indicate direct quotes from your manuscript), or elaborate on this item by providing additional information not in the ms, or briefly explain why the item is not applicable/relevant for your study

回答を入力

5) The interventions for each group with sufficient details to allow replication, including how and when they were actually administered

回答を編集しています。この URL を共有すると、他のユーザーもあなたの回答を編集できるようになります。 新しい回答を記入

5-i) Mention names, credential, affiliations of the developers, sponsors, and owners

Mention names, credential, affiliations of the developers, sponsors, and owners [6] (if authors/evaluators are owners or developer of the software, this needs to be declared in a “Conflict of interest” section or mentioned elsewhere in the manuscript).

	1	2	3	4	5	
subitem not at all important	○	○	○	○	○	essential

Does your paper address subitem 5-i?

Copy and paste relevant sections from the manuscript (include quotes in quotation marks "like this" to indicate direct quotes from your manuscript), or elaborate on this item by providing additional information not in the ms, or briefly explain why the item is not applicable/relevant for your study

回答を入力

5-ii) Describe the history/development process

Describe the history/development process of the application and previous formative evaluations (e.g., focus groups, usability testing), as these will have an impact on adoption/use rates and help with interpreting results.

	1	2	3	4	5	
subitem not at all important	○	○	○	○	○	essential

Does your paper address subitem 5-ii?

Copy and paste relevant sections from the manuscript (include quotes in quotation marks "like this" to indicate direct quotes from your manuscript), or elaborate on this item by providing additional information not in the ms, or briefly explain why the item is not applicable/relevant for your study

回答を入力

回答を編集しています。この URL を共有すると、他のユーザーもあなたの回答を編集できるようになります。

新しい回答を記入

5-iii) Revisions and updating

Revisions and updating. Clearly mention the date and/or version number of the application/intervention (and comparator, if applicable) evaluated, or describe whether the intervention underwent major changes during the evaluation process, or whether the development and/or content was “frozen” during the trial. Describe dynamic components such as news feeds or changing content which may have an impact on the replicability of the intervention (for unexpected events see item 3b).

	1	2	3	4	5	
subitem not at all important	○	○	○	○	○	essential

Does your paper address subitem 5-iii?

Copy and paste relevant sections from the manuscript (include quotes in quotation marks "like this" to indicate direct quotes from your manuscript), or elaborate on this item by providing additional information not in the ms, or briefly explain why the item is not applicable/relevant for your study

回答を入力

5-iv) Quality assurance methods

Provide information on quality assurance methods to ensure accuracy and quality of information provided [1], if applicable.

	1	2	3	4	5	
subitem not at all important	○	○	○	○	○	essential

Does your paper address subitem 5-iv?

Copy and paste relevant sections from the manuscript (include quotes in quotation marks "like this" to indicate direct quotes from your manuscript), or elaborate on this item by providing additional information not in the ms, or briefly explain why the item is not applicable/relevant for your study

回答を編集しています。この URL を共有すると、他のユーザーもあなたの回答を編集できるようになります。 新しい回答を記入

5-v) Ensure replicability by publishing the source code, and/or providing screenshots/screen-capture video, and/or providing flowcharts of the algorithms used

Ensure replicability by publishing the source code, and/or providing screenshots/screen-capture video, and/or providing flowcharts of the algorithms used. Replicability (i.e., other researchers should in principle be able to replicate the study) is a hallmark of scientific reporting.

	1	2	3	4	5	
subitem not at all important	○	○	○	○	○	essential

Does your paper address subitem 5-v?

Copy and paste relevant sections from the manuscript (include quotes in quotation marks "like this" to indicate direct quotes from your manuscript), or elaborate on this item by providing additional information not in the ms, or briefly explain why the item is not applicable/relevant for your study

回答を入力

5-vi) Digital preservation

Digital preservation: Provide the URL of the application, but as the intervention is likely to change or disappear over the course of the years; also make sure the intervention is archived (Internet Archive, webcitation.org, and/or publishing the source code or screenshots/videos alongside the article). As pages behind login screens cannot be archived, consider creating demo pages which are accessible without login.

	1	2	3	4	5	
subitem not at all important	○	○	○	○	○	essential

回答を編集しています。この URL を共有すると、他のユーザーもあなたの回答を編集できるようになります。 新しい回答を記入

Does your paper address subitem 5-vi?

Copy and paste relevant sections from the manuscript (include quotes in quotation marks "like this" to indicate direct quotes from your manuscript), or elaborate on this item by providing additional information not in the ms, or briefly explain why the item is not applicable/relevant for your study

回答を入力

5-vii) Access

Access: Describe how participants accessed the application, in what setting/context, if they had to pay (or were paid) or not, whether they had to be a member of specific group. If known, describe how participants obtained “access to the platform and Internet” [1]. To ensure access for editors/reviewers/readers, consider to provide a “backdoor” login account or demo mode for reviewers/readers to explore the application (also important for archiving purposes, see vi).

	1	2	3	4	5	
subitem not at all important	○	○	○	○	○	essential

Does your paper address subitem 5-vii? *

Copy and paste relevant sections from the manuscript (include quotes in quotation marks "like this" to indicate direct quotes from your manuscript), or elaborate on this item by providing additional information not in the ms, or briefly explain why the item is not applicable/relevant for your study

Participants evaluated clinical photographs of pressure ulcers sourced from two publications by the JSPU [23,24]. These publications are medically validated and authoritative sources that ensure the reliability and standardization of the referenced content. A total of 10 photographs were utilized, categorized into two sets (Questions 1–5 and Questions 6–10), with each set comprising five photographs (Table 1). The JSPU has formally provided permission for the utilization of these photographic materials in this investigation, contingent upon the requisite citation of the source.

回答を編集しています。この URL を共有すると、他のユーザーもあなたの回答を編集できるようになります。 新しい回答を記入

5-viii) Mode of delivery, features/functionalities/components of the intervention and comparator, and the theoretical framework

Describe mode of delivery, features/functionalities/components of the intervention and comparator, and the theoretical framework [6] used to design them (instructional strategy [1], behaviour change techniques, persuasive features, etc., see e.g., [7, 8] for terminology). This includes an in-depth description of the content (including where it is coming from and who developed it) [1],” whether [and how] it is tailored to individual circumstances and allows users to track their progress and receive feedback” [6]. This also includes a description of communication delivery channels and – if computer-mediated communication is a component – whether communication was synchronous or asynchronous [6]. It also includes information on presentation strategies [1], including page design principles, average amount of text on pages, presence of hyperlinks to other resources, etc. [1].

	1	2	3	4	5	
subitem not at all important	○	○	○	○	○	essential

Does your paper address subitem 5-viii? *

Copy and paste relevant sections from the manuscript (include quotes in quotation marks "like this" to indicate direct quotes from your manuscript), or elaborate on this item by providing additional information not in the ms, or briefly explain why the item is not applicable/relevant for your study

Group A first answered five multiple-choice questions as part of an intervention phase using the CDSS. After a washout period, the participants answered another five questions as part of a control phase without using the CDSS.

5-ix) Describe use parameters

Describe use parameters (e.g., intended “doses” and optimal timing for use). Clarify what instructions or recommendations were given to the user, e.g., regarding timing, frequency, heaviness of use, if any, or was the intervention used ad libitum.

	1	2	3	4	5	
subitem not at all important	○	○	○	○	○	essential

回答を編集しています。この URL を共有すると、他のユーザーもあなたの回答を編集できるようになります。 新しい回答を記入

Does your paper address subitem 5-ix?

Copy and paste relevant sections from the manuscript (include quotes in quotation marks "like this" to indicate direct quotes from your manuscript), or elaborate on this item by providing additional information not in the ms, or briefly explain why the item is not applicable/relevant for your study

回答を入力

5-x) Clarify the level of human involvement

Clarify the level of human involvement (care providers or health professionals, also technical assistance) in the e-intervention or as co-intervention (detail number and expertise of professionals involved, if any, as well as “type of assistance offered, the timing and frequency of the support, how it is initiated, and the medium by which the assistance is delivered”. It may be necessary to distinguish between the level of human involvement required for the trial, and the level of human involvement required for a routine application outside of a RCT setting (discuss under item 21 – generalizability).

	1	2	3	4	5	
subitem not at all important	○	○	○	○	○	essential

Does your paper address subitem 5-x?

Copy and paste relevant sections from the manuscript (include quotes in quotation marks "like this" to indicate direct quotes from your manuscript), or elaborate on this item by providing additional information not in the ms, or briefly explain why the item is not applicable/relevant for your study

回答を入力

回答を編集しています。この URL を共有すると、他のユーザーもあなたの回答を編集できるようになります。 新しい回答を記入

5-xi) Report any prompts/reminders used

Report any prompts/reminders used: Clarify if there were prompts (letters, emails, phone calls, SMS) to use the application, what triggered them, frequency etc. It may be necessary to distinguish between the level of prompts/reminders required for the trial, and the level of prompts/reminders for a routine application outside of a RCT setting (discuss under item 21 – generalizability).

	1	2	3	4	5	
subitem not at all important	○	○	○	○	○	essential

Does your paper address subitem 5-xi? *

Copy and paste relevant sections from the manuscript (include quotes in quotation marks "like this" to indicate direct quotes from your manuscript), or elaborate on this item by providing additional information not in the ms, or briefly explain why the item is not applicable/relevant for your study

For this study, a prototype CDSS was created using the web-based no-code platform Canva [15]. The CDSS is a prototype of a web application designed for use on mobile devices, such as smartphones. The system has been developed to provide a framework for decision-making regarding the selection of ointments and wound dressings, through a series of questions related to the patient's pressure ulcers, as illustrated in Figure 1. Figure 2 presents an example of option selection in the CDSS. The system's purpose is to supplement physicians' decision-making in various clinical settings, including the GIM setting. Two existing clinical guidelines were utilized to support Japanese physicians: the Guidelines for the Diagnosis and Treatment of Pressure Ulcers published by the Japanese Dermatological Association and the Guidelines for the Prevention and Management of Pressure Ulcers published by the Japanese Society of Pressure Ulcers (JSPU) [16,17].

5-xii) Describe any co-interventions (incl. training/support)

Describe any co-interventions (incl. training/support): Clearly state any interventions that are provided in addition to the targeted eHealth intervention, as ehealth intervention may not be designed as stand-alone intervention. This includes training sessions and support [1]. It may be necessary to distinguish between the level of training required for the trial, and the level of training for a routine application outside of a RCT setting (discuss under item 21 – generalizability.

1	2	3	4	5

回答を編集しています。この URL を共有すると、他のユーザーもあなたの回答を編集できるようになります。 新しい回答を記入

Does your paper address subitem 5-xii? *

Copy and paste relevant sections from the manuscript (include quotes in quotation marks "like this" to indicate direct quotes from your manuscript), or elaborate on this item by providing additional information not in the ms, or briefly explain why the item is not applicable/relevant for your study

This research was not any co-interventions (incl. training/support).

6a) Completely defined pre-specified primary and secondary outcome measures, including how and when they were assessed

Does your paper address CONSORT subitem 6a? *

Copy and paste relevant sections from the manuscript (include quotes in quotation marks "like this" to indicate direct quotes from your manuscript), or elaborate on this item by providing additional information not in the ms, or briefly explain why the item is not applicable/relevant for your study

The primary outcome was the proportion of participants who selected the correct answers for both ointment and wound dressing options. The secondary outcomes were comparisons of the correct response rates for ointment selections and those for wound dressing selections.

6a-i) Online questionnaires: describe if they were validated for online use and apply CHERRIES items to describe how the questionnaires were designed/deployed

If outcomes were obtained through online questionnaires, describe if they were validated for online use and apply CHERRIES items to describe how the questionnaires were designed/deployed [9].

	1	2	3	4	5	
subitem not at all important	○	○	○	○	○	essential

回答を編集しています。この URL を共有すると、他のユーザーもあなたの回答を編集できるようになります。 新しい回答を記入

Does your paper address subitem 6a-i?

Copy and paste relevant sections from manuscript text

回答を入力

6a-ii) Describe whether and how “use” (including intensity of use/dosage) was defined/measured/monitored

Describe whether and how “use” (including intensity of use/dosage) was defined/measured/monitored (logins, logfile analysis, etc.). Use/adoption metrics are important process outcomes that should be reported in any ehealth trial.

	1	2	3	4	5	
subitem not at all important	○	○	○	○	○	essential

Does your paper address subitem 6a-ii?

Copy and paste relevant sections from manuscript text

回答を入力

6a-iii) Describe whether, how, and when qualitative feedback from participants was obtained

Describe whether, how, and when qualitative feedback from participants was obtained (e.g., through emails, feedback forms, interviews, focus groups).

	1	2	3	4	5	
subitem not at all important	○	○	○	○	○	essential

回答を編集しています。この URL を共有すると、他のユーザーもあなたの回答を編集できるようになります。 新しい回答を記入

Does your paper address subitem 6a-iii?

Copy and paste relevant sections from manuscript text

回答を入力

6b) Any changes to trial outcomes after the trial commenced, with reasons

Does your paper address CONSORT subitem 6b? *

Copy and paste relevant sections from the manuscript (include quotes in quotation marks "like this" to indicate direct quotes from your manuscript), or elaborate on this item by providing additional information not in the ms, or briefly explain why the item is not applicable/relevant for your study

This study was not changed to trial outcomes.

7a) How sample size was determined

NPT: When applicable, details of whether and how the clustering by care provides or centers was addressed

7a-i) Describe whether and how expected attrition was taken into account when calculating the sample size

Describe whether and how expected attrition was taken into account when calculating the sample size.

	1	2	3	4	5	
subitem not at all important	○	○	○	○	○	essential

回答を編集しています。この URL を共有すると、他のユーザーもあなたの回答を編集できるようになります。

新しい回答を記入

Does your paper address subitem 7a-i?

Copy and paste relevant sections from manuscript title (include quotes in quotation marks "like this" to indicate direct quotes from your manuscript), or elaborate on this item by providing additional information not in the ms, or briefly explain why the item is not applicable/relevant for your study

回答を入力

7b) When applicable, explanation of any interim analyses and stopping guidelines

Does your paper address CONSORT subitem 7b? *

Copy and paste relevant sections from the manuscript (include quotes in quotation marks "like this" to indicate direct quotes from your manuscript), or elaborate on this item by providing additional information not in the ms, or briefly explain why the item is not applicable/relevant for your study

This study was not included explanation of any interim analyses and stopping guidelines.

8a) Method used to generate the random allocation sequence

NPT: When applicable, how care providers were allocated to each trial group

Does your paper address CONSORT subitem 8a? *

Copy and paste relevant sections from the manuscript (include quotes in quotation marks "like this" to indicate direct quotes from your manuscript), or elaborate on this item by providing additional information not in the ms, or briefly explain why the item is not applicable/relevant for your study

The randomization process was conducted electronically using Microsoft Excel (Microsoft, Redmond, WA, USA) and a computer-generated randomization table. Using block randomization with the sealed envelope method, participants were randomly assigned to Group A or B in a 1:1 ratio.

回答を編集しています。この URL を共有すると、他のユーザーもあなたの回答を編集できるようになります。 新しい回答を記入

Does your paper address CONSORT subitem 8b? *

Copy and paste relevant sections from the manuscript (include quotes in quotation marks "like this" to indicate direct quotes from your manuscript), or elaborate on this item by providing additional information not in the ms, or briefly explain why the item is not applicable/relevant for your study

The randomization process was conducted electronically using Microsoft Excel (Microsoft, Redmond, WA, USA) and a computer-generated randomization table. Using block randomization with the sealed envelope method, participants were randomly assigned to Group A or B in a 1:1 ratio.

9) Mechanism used to implement the random allocation sequence (such as sequentially numbered containers), describing any steps taken to conceal the sequence until interventions were assigned

Does your paper address CONSORT subitem 9? *

Copy and paste relevant sections from the manuscript (include quotes in quotation marks "like this" to indicate direct quotes from your manuscript), or elaborate on this item by providing additional information not in the ms, or briefly explain why the item is not applicable/relevant for your study

The randomization process was conducted electronically using Microsoft Excel (Microsoft, Redmond, WA, USA) and a computer-generated randomization table. Using block randomization with the sealed envelope method, participants were randomly assigned to Group A or B in a 1:1 ratio.

10) Who generated the random allocation sequence, who enrolled participants, and who assigned participants to interventions

回答を編集しています。この URL を共有すると、他のユーザーもあなたの回答を編集できるようになります。 新しい回答を記入

Does your paper address CONSORT subitem 10? *

Copy and paste relevant sections from the manuscript (include quotes in quotation marks "like this" to indicate direct quotes from your manuscript), or elaborate on this item by providing additional information not in the ms, or briefly explain why the item is not applicable/relevant for your study

The random allocation sequence was generated by an independent researcher, TH, using Microsoft Excel.

11a) If done, who was blinded after assignment to interventions (for example, participants, care providers, those assessing outcomes) and how

NPT: Whether or not administering co-interventions were blinded to group assignment

11a-i) Specify who was blinded, and who wasn't

Specify who was blinded, and who wasn't. Usually, in web-based trials it is not possible to blind the participants [1, 3] (this should be clearly acknowledged), but it may be possible to blind outcome assessors, those doing data analysis or those administering co-interventions (if any).

	1	2	3	4	5	
subitem not at all important	○	○	○	○	○	essential

Does your paper address subitem 11a-i? *

Copy and paste relevant sections from the manuscript (include quotes in quotation marks "like this" to indicate direct quotes from your manuscript), or elaborate on this item by providing additional information not in the ms, or briefly explain why the item is not applicable/relevant for your study

This study was not included specify who was blinded, and who wasn't.

回答を編集しています。この URL を共有すると、他のユーザーもあなたの回答を編集できるようになります。 新しい回答を記入

11a-ii) Discuss e.g., whether participants knew which intervention was the "intervention of interest" and which one was the "comparator"

Informed consent procedures (4a-ii) can create biases and certain expectations - discuss e.g., whether participants knew which intervention was the "intervention of interest" and which one was the "comparator".

	1	2	3	4	5	
subitem not at all important	○	○	○	○	○	essential

Does your paper address subitem 11a-ii?

Copy and paste relevant sections from the manuscript (include quotes in quotation marks "like this" to indicate direct quotes from your manuscript), or elaborate on this item by providing additional information not in the ms, or briefly explain why the item is not applicable/relevant for your study

回答を入力

11b) If relevant, description of the similarity of interventions
(this item is usually not relevant for ehealth trials as it refers to similarity of a placebo or sham intervention to a active medication/intervention)

Does your paper address CONSORT subitem 11b? *

Copy and paste relevant sections from the manuscript (include quotes in quotation marks "like this" to indicate direct quotes from your manuscript), or elaborate on this item by providing additional information not in the ms, or briefly explain why the item is not applicable/relevant for your study

This study was not included of the similarity of interventions.

12a) Statistical methods used to compare groups for primary and secondary outcomes

NPT: When applicable, details of whether and how the clustering by care providers or [illegible]

回答を編集しています。この URL を共有すると、他のユーザーもあなたの回答を編集できるようになります。

新しい回答を記入

Does your paper address CONSORT subitem 12a? *

Copy and paste relevant sections from the manuscript (include quotes in quotation marks "like this" to indicate direct quotes from your manuscript), or elaborate on this item by providing additional information not in the ms, or briefly explain why the item is not applicable/relevant for your study

Statistical analyses were performed using R version 4.5.0 for Windows (The R Foundation for Statistical Computing, Vienna, Austria). Statistical significance was set at P<.05, and 95% confidence intervals (CIs) were provided in addition to p-values. The correct response rates in each group were compared using Fisher’s exact test for primary and secondary outcome measures. The Mann−Whitney U test was used to compare continuous variables for baseline participant characteristics, which are presented as medians (IQRs). Fisher’s exact test was used to compare the categorical and binary variables for baseline participant characteristics, which are presented as counts (percentages).

12a-i) Imputation techniques to deal with attrition / missing values

Imputation techniques to deal with attrition / missing values: Not all participants will use the intervention/comparator as intended and attrition is typically high in ehealth trials. Specify how participants who did not use the application or dropped out from the trial were treated in the statistical analysis (a complete case analysis is strongly discouraged, and simple imputation techniques such as LOCF may also be problematic [4]).

	1	2	3	4	5	
subitem not at all important	○	○	○	○	○	essential

Does your paper address subitem 12a-i? *

Copy and paste relevant sections from the manuscript (include quotes in quotation marks "like this" to indicate direct quotes from your manuscript), or elaborate on this item by providing additional information not in the ms, or briefly explain why the item is not applicable/relevant for your study

This study was not included missing values.

12b) Methods for additional analyses, such as subgroup analyses and adjusted

回答を編集しています。この URL を共有すると、他のユーザーもあなたの回答を編集できるようになります。 新しい回答を記入

Does your paper address CONSORT subitem 12b? *

Copy and paste relevant sections from the manuscript (include quotes in quotation marks "like this" to indicate direct quotes from your manuscript), or elaborate on this item by providing additional information not in the ms, or briefly explain why the item is not applicable/relevant for your study

The secondary outcome measures were comparisons of the correct response rates for ointment selections and those for wound dressing selections.

X26) REB/IRB Approval and Ethical Considerations [recommended as subheading under "Methods"] (not a CONSORT item)

X26-i) Comment on ethics committee approval

	1	2	3	4	5	
subitem not at all important	○	○	○	○	○	essential

Does your paper address subitem X26-i?

Copy and paste relevant sections from the manuscript (include quotes in quotation marks "like this" to indicate direct quotes from your manuscript), or elaborate on this item by providing additional information not in the ms, or briefly explain why the item is not applicable/relevant for your study

回答を入力

x26-ii) Outline informed consent procedures

Outline informed consent procedures e.g., if consent was obtained offline or online (how? Checkbox, etc.?), and what information was provided (see 4a-ii). See [6] for some items to be included in informed consent documents.

1 2 3 4 5

回答を編集しています。この URL を共有すると、他のユーザーもあなたの回答を編集できるようになります。

新しい回答を記入

Does your paper address subitem X26-ii?

Copy and paste relevant sections from the manuscript (include quotes in quotation marks "like this" to indicate direct quotes from your manuscript), or elaborate on this item by providing additional information not in the ms, or briefly explain why the item is not applicable/relevant for your study

回答を入力

X26-iii) Safety and security procedures

Safety and security procedures, incl. privacy considerations, and any steps taken to reduce the likelihood or detection of harm (e.g., education and training, availability of a hotline)

	1	2	3	4	5	
subitem not at all important	○	○	○	○	○	essential

Does your paper address subitem X26-iii?

Copy and paste relevant sections from the manuscript (include quotes in quotation marks "like this" to indicate direct quotes from your manuscript), or elaborate on this item by providing additional information not in the ms, or briefly explain why the item is not applicable/relevant for your study

回答を入力

RESULTS

13a) For each group, the numbers of participants who were randomly assigned, received intended treatment, and were analysed for the primary outcome

NPT: The number of care providers or centers performing the intervention in each group and the number of patients treated by each care provider in each center

回答を編集しています。この URL を共有すると、他のユーザーもあなたの回答を編集できるようになります。 新しい回答を記入

Does your paper address CONSORT subitem 13a? *

Copy and paste relevant sections from the manuscript (include quotes in quotation marks "like this" to indicate direct quotes from your manuscript), or elaborate on this item by providing additional information not in the ms, or briefly explain why the item is not applicable/relevant for your study

The primary outcome measure was the proportion of participants who correctly answered both the ointment and wound dressing selections. A total of twenty-eight participants answered five questions during the intervention phase and other five during the control phase. As a result, 140 responses were collected for each phase, calculated as 28 participants × 5 questions.

13b) For each group, losses and exclusions after randomisation, together with reasons

Does your paper address CONSORT subitem 13b? (NOTE: Preferably, this is shown in a CONSORT flow diagram) *

Copy and paste relevant sections from the manuscript (include quotes in quotation marks "like this" to indicate direct quotes from your manuscript), or elaborate on this item by providing additional information not in the ms, or briefly explain why the item is not applicable/relevant for your study

This study was not included losses and exclusions after randomisation.

13b-i) Attrition diagram

Strongly recommended: An attrition diagram (e.g., proportion of participants still logging in or using the intervention/comparator in each group plotted over time, similar to a survival curve) or other figures or tables demonstrating usage/dose/engagement.

	1	2	3	4	5	
subitem not at all important	○	○	○	○	○	essential

回答を編集しています。この URL を共有すると、他のユーザーもあなたの回答を編集できるようになります。 新しい回答を記入

Does your paper address subitem 13b-i?

Copy and paste relevant sections from the manuscript or cite the figure number if applicable (include quotes in quotation marks "like this" to indicate direct quotes from your manuscript), or elaborate on this item by providing additional information not in the ms, or briefly explain why the item is not applicable/relevant for your study

回答を入力

14a) Dates defining the periods of recruitment and follow-up

Does your paper address CONSORT subitem 14a? *

Copy and paste relevant sections from the manuscript (include quotes in quotation marks "like this" to indicate direct quotes from your manuscript), or elaborate on this item by providing additional information not in the ms, or briefly explain why the item is not applicable/relevant for your study

This study was conducted by the Department of Diagnostic and Generalist Medicine (the Department of GIM) at Dokkyo Medical University Hospital, Tochigi, Japan, in March 2025 and May 2025.

14a-i) Indicate if critical “secular events” fell into the study period

Indicate if critical “secular events” fell into the study period, e.g., significant changes in Internet resources available or “changes in computer hardware or Internet delivery resources”

	1	2	3	4	5	
subitem not at all important	○	○	○	○	○	essential

回答を編集しています。この URL を共有すると、他のユーザーもあなたの回答を編集できるようになります。 新しい回答を記入

Does your paper address subitem 14a-i?

Copy and paste relevant sections from the manuscript (include quotes in quotation marks "like this" to indicate direct quotes from your manuscript), or elaborate on this item by providing additional information not in the ms, or briefly explain why the item is not applicable/relevant for your study

回答を入力

14b) Why the trial ended or was stopped (early)

Does your paper address CONSORT subitem 14b? *

Copy and paste relevant sections from the manuscript (include quotes in quotation marks "like this" to indicate direct quotes from your manuscript), or elaborate on this item by providing additional information not in the ms, or briefly explain why the item is not applicable/relevant for your study

This study was ended after enrolled required sample size.

15) A table showing baseline demographic and clinical characteristics for each group

NPT: When applicable, a description of care providers (case volume, qualification, expertise, etc.) and centers (volume) in each group

Does your paper address CONSORT subitem 15? *

Copy and paste relevant sections from the manuscript (include quotes in quotation marks "like this" to indicate direct quotes from your manuscript), or elaborate on this item by providing additional information not in the ms, or briefly explain why the item is not applicable/relevant for your study

A total of 28 GIM physicians participated in this study. The median age of all participants was 32.2 (IQR 7.0) years, 20 participants (71.4%) were male, and the median number of years since graduation was 7.3 (IQR 6.0). Participant age (P=.39), sex (P=.21), and years since graduation (P=.25) did not differ significantly between Group A and Group B (Table 2).

回答を編集しています。この URL を共有すると、他のユーザーもあなたの回答を編集できるようになります。 新しい回答を記入

15-i) Report demographics associated with digital divide issues

In ehealth trials it is particularly important to report demographics associated with digital divide issues, such as age, education, gender, social-economic status, computer/Internet/ehealth literacy of the participants, if known.

	1	2	3	4	5	
subitem not at all important	○	○	○	○	○	essential

Does your paper address subitem 15-i? *

Copy and paste relevant sections from the manuscript (include quotes in quotation marks "like this" to indicate direct quotes from your manuscript), or elaborate on this item by providing additional information not in the ms, or briefly explain why the item is not applicable/relevant for your study

A total of 28 GIM physicians participated in this study. The median age of all participants was 32.2 (IQR 7.0) years, 20 participants (71.4%) were male, and the median number of years since graduation was 7.3 (IQR 6.0). Participant age (P=.39), sex (P=.21), and years since graduation (P=.25) did not differ significantly between Group A and Group B (Table 2).

16) For each group, number of participants (denominator) included in each analysis and whether the analysis was by original assigned groups

16-i) Report multiple “denominators” and provide definitions

Report multiple “denominators” and provide definitions: Report N’s (and effect sizes) “across a range of study participation [and use] thresholds” [1], e.g., N exposed, N consented, N used more than x times, N used more than y weeks, N participants “used” the intervention/comparator at specific pre-defined time points of interest (in absolute and relative numbers per group). Always clearly define “use” of the intervention.

	1	2	3	4	5	
subitem not at all important	○	○	○	○	○	essential

回答を編集しています。この URL を共有すると、他のユーザーもあなたの回答を編集できるようになります。 新しい回答を記入

Does your paper address subitem 16-i? *

Copy and paste relevant sections from the manuscript (include quotes in quotation marks "like this" to indicate direct quotes from your manuscript), or elaborate on this item by providing additional information not in the ms, or briefly explain why the item is not applicable/relevant for your study

A total of 28 GIM physicians participated in this study. The median age of all participants was 32.2 (IQR 7.0) years, 20 participants (71.4%) were male, and the median number of years since graduation was 7.3 (IQR 6.0). Participant age (P=.39), sex (P=.21), and years since graduation (P=.25) did not differ significantly between Group A and Group B (Table 2).

16-ii) Primary analysis should be intent-to-treat

Primary analysis should be intent-to-treat, secondary analyses could include comparing only “users”, with the appropriate caveats that this is no longer a randomized sample (see 18-i).

	1	2	3	4	5	
subitem not at all important	○	○	○	○	○	essential

Does your paper address subitem 16-ii?

Copy and paste relevant sections from the manuscript (include quotes in quotation marks "like this" to indicate direct quotes from your manuscript), or elaborate on this item by providing additional information not in the ms, or briefly explain why the item is not applicable/relevant for your study

回答を入力

17a) For each primary and secondary outcome, results for each group, and the estimated effect size and its precision (such as 95% confidence interval)

回答を編集しています。この URL を共有すると、他のユーザーもあなたの回答を編集できるようになります。

新しい回答を記入

Does your paper address CONSORT subitem 17a? *

Copy and paste relevant sections from the manuscript (include quotes in quotation marks "like this" to indicate direct quotes from your manuscript), or elaborate on this item by providing additional information not in the ms, or briefly explain why the item is not applicable/relevant for your study

The primary outcome was the proportion of participants who correctly selected both the most appropriate ointment and wound dressing options (Table 3). The overall correct response rates were 49.3% (69/140 [95% CI: 41.1%–57.5%]) in the intervention phase and 5.0% (7/140 [95% CI: 2.4%–10.0%]) in the control phase, with a significant difference between groups (P<.001). A comparison of the correct response rates between the intervention phase and the control phase for each question is listed in Multimedia Appendix 1.

The secondary outcomes were comparisons of the correct response rates for ointment and those for wound dressing (Table 3). The correct response rates for ointment were 65.0% (91/140 [95% CI: 56.8%–72.4%]) in the intervention phase and 45.0% (63/140 [95% CI: 37.0%–53.3%]) in the control phase (P=.001). The correct response rates for wound dressing were 59.3% (83/140 [95% CI: 51.0%–67.1%]) in the intervention group and 15.0% (21/140 [95% CI: 10.0%–21.8%]) in the control group, with a significant difference between groups (P<.001). A comparison of the correct response rates between the intervention phase and the control phase for each question is listed in Multimedia Appendix 2 and 3.

17a-i) Presentation of process outcomes such as metrics of use and intensity of use

In addition to primary/secondary (clinical) outcomes, the presentation of process outcomes such as metrics of use and intensity of use (dose, exposure) and their operational definitions is critical. This does not only refer to metrics of attrition (13-b) (often a binary variable), but also to more continuous exposure metrics such as “average session length”. These must be accompanied by a technical description how a metric like a “session” is defined (e.g., timeout after idle time) [1] (report under item 6a).

	1	2	3	4	5	
subitem not at all important	○	○	○	○	○	essential

回答を編集しています。この URL を共有すると、他のユーザーもあなたの回答を編集できるようになります。 新しい回答を記入

Does your paper address subitem 17a-i?

Copy and paste relevant sections from the manuscript (include quotes in quotation marks "like this" to indicate direct quotes from your manuscript), or elaborate on this item by providing additional information not in the ms, or briefly explain why the item is not applicable/relevant for your study

回答を入力

17b) For binary outcomes, presentation of both absolute and relative effect sizes is recommended

Does your paper address CONSORT subitem 17b? *

Copy and paste relevant sections from the manuscript (include quotes in quotation marks "like this" to indicate direct quotes from your manuscript), or elaborate on this item by providing additional information not in the ms, or briefly explain why the item is not applicable/relevant for your study

There was no binary outcomes in this study.

18) Results of any other analyses performed, including subgroup analyses and adjusted analyses, distinguishing pre-specified from exploratory

Does your paper address CONSORT subitem 18? *

Copy and paste relevant sections from the manuscript (include quotes in quotation marks "like this" to indicate direct quotes from your manuscript), or elaborate on this item by providing additional information not in the ms, or briefly explain why the item is not applicable/relevant for your study

An exploratory analysis was conducted to compare correct response rates in the intervention and control phases for the pressure ulcer categories “Deep” and “Non-deep” (Table 5). The correct response rates for “Deep” pressure ulcer were 49.0% (48/98 [95% CI: 39.3%−58.7%]) in the intervention group and 2.0% (2/98 [95% CI: 0.6%−7.1%]) in the control group, with a significant difference between groups (P<.001). The correct response rates for “Non-deep” pressure ulcer were 54.8% (23/42 [95% CI: 39.9%−68.8%]) in the intervention

回答を編集しています。この URL を共有すると、他のユーザーもあなたの回答を編集できるようになります。 新しい回答を記入

18-i) Subgroup analysis of comparing only users

A subgroup analysis of comparing only users is not uncommon in ehealth trials, but if done, it must be stressed that this is a self-selected sample and no longer an unbiased sample from a randomized trial (see 16-iii).

	1	2	3	4	5	
subitem not at all important	○	○	○	○	○	essential

Does your paper address subitem 18-i?

Copy and paste relevant sections from the manuscript (include quotes in quotation marks "like this" to indicate direct quotes from your manuscript), or elaborate on this item by providing additional information not in the ms, or briefly explain why the item is not applicable/relevant for your study

回答を入力

19) All important harms or unintended effects in each group

(for specific guidance see CONSORT for harms)

Does your paper address CONSORT subitem 19? *

Copy and paste relevant sections from the manuscript (include quotes in quotation marks "like this" to indicate direct quotes from your manuscript), or elaborate on this item by providing additional information not in the ms, or briefly explain why the item is not applicable/relevant for your study

This research was included all important harms or unintended effects in each group.

回答を編集しています。この URL を共有すると、他のユーザーもあなたの回答を編集できるようになります。 新しい回答を記入

19-i) Include privacy breaches, technical problems

Include privacy breaches, technical problems. This does not only include physical “harm” to participants, but also incidents such as perceived or real privacy breaches [1], technical problems, and other unexpected/unintended incidents. “Unintended effects” also includes unintended positive effects [2].

	1	2	3	4	5	
subitem not at all important	○	○	○	○	○	essential

Does your paper address subitem 19-i?

Copy and paste relevant sections from the manuscript (include quotes in quotation marks "like this" to indicate direct quotes from your manuscript), or elaborate on this item by providing additional information not in the ms, or briefly explain why the item is not applicable/relevant for your study

回答を入力

19-ii) Include qualitative feedback from participants or observations from staff/researchers

Include qualitative feedback from participants or observations from staff/researchers, if available, on strengths and shortcomings of the application, especially if they point to unintended/unexpected effects or uses. This includes (if available) reasons for why people did or did not use the application as intended by the developers.

	1	2	3	4	5	
subitem not at all important	○	○	○	○	○	essential

Does your paper address subitem 19-ii?

Copy and paste relevant sections from the manuscript (include quotes in quotation marks "like this" to indicate direct quotes from your manuscript), or elaborate on this item by providing additional information not in the ms, or briefly explain why the item is not applicable/relevant for your study

回答を編集しています。この URL を共有すると、他のユーザーもあなたの回答を編集できるようになります。

新しい回答を記入

DISCUSSION

22) Interpretation consistent with results, balancing benefits and harms, and considering other relevant evidence

NPT: In addition, take into account the choice of the comparator, lack of or partial blinding, and unequal expertise of care providers or centers in each group

22-i) Restate study questions and summarize the answers suggested by the data, starting with primary outcomes and process outcomes (use)

Restate study questions and summarize the answers suggested by the data, starting with primary outcomes and process outcomes (use).

	1	2	3	4	5	
subitem not at all important	○	○	○	○	○	essential

回答を編集しています。この URL を共有すると、他のユーザーもあなたの回答を編集できるようになります。 新しい回答を記入

Does your paper address subitem 22-i? *

Copy and paste relevant sections from the manuscript (include quotes in quotation marks "like this" to indicate direct quotes from your manuscript), or elaborate on this item by providing additional information not in the ms, or briefly explain why the item is not applicable/relevant for your study

This study yielded three main findings. First, the use of the CDSS increased the correct response rates by GIM physicians for both ointment and wound dressings (49.3% [69/140] vs. 5.0% [7/140], P<.001). The correct response rates in the control phase were extremely low; selecting both the appropriate ointment and dressing to treat pressure ulcers was challenging. This result suggests that the CDSS has the potential to assist physicians in cases of uncertainty about the appropriate treatment for pressure ulcers.

The secondary outcome measures were comparisons of the correct response rates for ointment selection and those for wound dressing selection, which showed significantly higher rates when using the CDSS (ointment: 65.0% [91/140] vs. 45.0% [63/140], P=.001; wound dressings: 59.3% [83/140] vs. 15.0% [21/140], P<.001). Notably, the CDSS was particularly useful in the selection of the most appropriate wound dressings. Some physicians reportedly lack knowledge about wound dressing properties, such as their application according to the stage of the ulcer [25]. Therefore, assistance in selecting wound dressings is likely to impact clinical practice in settings where consultation with specialists is not available immediately.

The third major finding is that the CDSS may have contributed to the improvement in correct response rates for both “Deep” and “Non-deep” pressure ulcers. Although the number of questions on ulcer depth was small, the consistently higher correct response rates in the intervention phase suggest that the CDSS facilitated the identification of both “Deep” and “Non-deep” ulcers.

22-ii) Highlight unanswered new questions, suggest future research

Highlight unanswered new questions, suggest future research.

	1	2	3	4	5	
subitem not at all important	○	○	○	○	○	essential

回答を編集しています。この URL を共有すると、他のユーザーもあなたの回答を編集できるようになります。 新しい回答を記入

Does your paper address subitem 22-ii?

Copy and paste relevant sections from the manuscript (include quotes in quotation marks "like this" to indicate direct quotes from your manuscript), or elaborate on this item by providing additional information not in the ms, or briefly explain why the item is not applicable/relevant for your study

回答を入力

20) Trial limitations, addressing sources of potential bias, imprecision, and, if relevant, multiplicity of analyses

20-i) Typical limitations in ehealth trials

Typical limitations in ehealth trials: Participants in ehealth trials are rarely blinded. Ehealth trials often look at a multiplicity of outcomes, increasing risk for a Type I error. Discuss biases due to non-use of the intervention/usability issues, biases through informed consent procedures, unexpected events.

	1	2	3	4	5	
subitem not at all important	○	○	○	○	○	essential

回答を編集しています。この URL を共有すると、他のユーザーもあなたの回答を編集できるようになります。

新しい回答を記入

Does your paper address subitem 20-i? *

Copy and paste relevant sections from the manuscript (include quotes in quotation marks "like this" to indicate direct quotes from your manuscript), or elaborate on this item by providing additional information not in the ms, or briefly explain why the item is not applicable/relevant for your study

This was a pilot study with three limitations. First, the study's methodology was confined to the analysis of clinical photographs of pressure ulcers, excluding clinical courses, other physical examinations, and investigation results from a real-world setting. Consequently, to substantiate the efficacy and legitimacy of the CDSS for pressure ulcer management in clinical practice, subsequent studies involving actual patients, with careful consideration for patient safety, are required.

Second, this study used a crossover design with a washout period between phases. Consequently, there remains a possibility of bias from learning or carryover effects. However, sensitivity analysis indicated that a significant difference remained between the intervention and control groups even when comparing correct response rates in the first phase alone, suggesting that such bias is likely minimal.

Third, the sample size was relatively small. This study was conducted by a department of GIM at a single institution. In the future, studies should include a more diverse sample of the target population. For example, home-visit physicians and physicians from other specialties working in the inpatient wards should be included. In addition, decision-making by other healthcare professionals is a critical element in preventing and managing pressure ulcers [26]. CDSSs for pressure ulcer prevention and treatment may assist nurses in effectively reducing the incidence of pressure ulcers [27]. Therefore, nurses may also consider the use of the system.

21) Generalisability (external validity, applicability) of the trial findings

NPT: External validity of the trial findings according to the intervention, comparators, patients, and care providers or centers involved in the trial

21-i) Generalizability to other populations

Generalizability to other populations: In particular, discuss generalizability to a general Internet population, outside of a RCT setting, and general patient population, including applicability of the study results for other organizations

	1	2	3	4	5	
subitem not at all important	○	○	○	○	○	essential

回答を編集しています。この URL を共有すると、他のユーザーもあなたの回答を編集できるようになります。 新しい回答を記入

Does your paper address subitem 21-i?

Copy and paste relevant sections from the manuscript (include quotes in quotation marks "like this" to indicate direct quotes from your manuscript), or elaborate on this item by providing additional information not in the ms, or briefly explain why the item is not applicable/relevant for your study

回答を入力

21-ii) Discuss if there were elements in the RCT that would be different in a routine application setting

Discuss if there were elements in the RCT that would be different in a routine application setting (e.g., prompts/reminders, more human involvement, training sessions or other co-interventions) and what impact the omission of these elements could have on use, adoption, or outcomes if the intervention is applied outside of a RCT setting.

	1	2	3	4	5	
subitem not at all important	○	○	○	○	○	essential

Does your paper address subitem 21-ii?

Copy and paste relevant sections from the manuscript (include quotes in quotation marks "like this" to indicate direct quotes from your manuscript), or elaborate on this item by providing additional information not in the ms, or briefly explain why the item is not applicable/relevant for your study

回答を入力

OTHER INFORMATION

23) Registration number and name of trial registry

回答を編集しています。この URL を共有すると、他のユーザーもあなたの回答を編集できるようになります。 新しい回答を記入

Does your paper address CONSORT subitem 23? *

Copy and paste relevant sections from the manuscript (include quotes in quotation marks "like this" to indicate direct quotes from your manuscript), or elaborate on this item by providing additional information not in the ms, or briefly explain why the item is not applicable/relevant for your study

Trial Registration: UMIN Clinical Trials Registry (UMIN000057294) https://center6.umin.ac.jp/cgi-open-bin/ctr_e/ctr_view.cgi?recptno=R000064898

24) Where the full trial protocol can be accessed, if available

Does your paper address CONSORT subitem 24? *

Cite a Multimedia Appendix, other reference, or copy and paste relevant sections from the manuscript (include quotes in quotation marks "like this" to indicate direct quotes from your manuscript), or elaborate on this item by providing additional information not in the ms, or briefly explain why the item is not applicable/relevant for your study

Trial Registration: UMIN Clinical Trials Registry (UMIN000057294) https://center6.umin.ac.jp/cgi-open-bin/ctr_e/ctr_view.cgi?recptno=R000064898

25) Sources of funding and other support (such as supply of drugs), role of funders

Does your paper address CONSORT subitem 25? *

Copy and paste relevant sections from the manuscript (include quotes in quotation marks "like this" to indicate direct quotes from your manuscript), or elaborate on this item by providing additional information not in the ms, or briefly explain why the item is not applicable/relevant for your study

There was no funding to disclose for this study.

X27) Conflicts of Interest (not a CONSORT item)

回答を編集しています。この URL を共有すると、他のユーザーもあなたの回答を編集できるようになります。 新しい回答を記入

X27-i) State the relation of the study team towards the system being evaluated

In addition to the usual declaration of interests (financial or otherwise), also state the relation of the study team towards the system being evaluated, i.e., state if the authors/evaluators are distinct from or identical with the developers/sponsors of the intervention.

	1	2	3	4	5	
subitem not at all important	○	○	○	○	○	essential

Does your paper address subitem X27-i?

Copy and paste relevant sections from the manuscript (include quotes in quotation marks "like this" to indicate direct quotes from your manuscript), or elaborate on this item by providing additional information not in the ms, or briefly explain why the item is not applicable/relevant for your study

回答を入力

About the CONSORT EHEALTH checklist

As a result of using this checklist, did you make changes in your manuscript? *

- ○ yes, major changes
- ○ yes, minor changes
- ◉ no

What were the most important changes you made as a result of using this checklist?

回答を入力

回答を編集しています。この URL を共有すると、他のユーザーもあなたの回答を編集できるようになります。 新しい回答を記入

How much time did you spend on going through the checklist INCLUDING making changes in your manuscript *

We spent more than 30 minutes.

As a result of using this checklist, do you think your manuscript has improved? *

- ○ yes
- ◉ no
- ○ その他:

Would you like to become involved in the CONSORT EHEALTH group?

This would involve for example becoming involved in participating in a workshop and writing an "Explanation and Elaboration" document

- ○ yes
- ○ no
- ○ その他:

Any other comments or questions on CONSORT EHEALTH

回答を入力

STOP - Save this form as PDF before you click submit

To generate a record that you filled in this form, we recommend to generate a PDF of this page (on a Mac, simply select "print" and then select "print as PDF") before you submit it.

When you submit your (revised) paper to JMIR, please upload the PDF as supplementary file.

回答を編集しています。この URL を共有すると、他のユーザーもあなたの回答を編集できるようになります。 新しい回答を記入

Final step: Click submit !

Click submit so we have your answers in our database!

送信

Google フォームでパスワードを送信しないでください。

このコンテンツは Google が作成または承認したものではありません。 - フォームのオーナーに問い合わせる - 利用規約 - プライバシー ポリシー

このフォームが不審だと思われる場合 報告

Google フォーム

回答を編集しています。この URL を共有すると、他のユーザーもあなたの回答を編集できるようになります。

新しい回答を記入

回答を編集しています。この URL を共有すると、他のユーザーもあなたの回答を編集できるようになります。

新しい回答を記入
